# Accelerated biological aging and risk of sarcopenia: evidence from 29,000 Chinese adults

**DOI:** 10.5114/biolsport.2026.159572

**Published:** 2026-04-13

**Authors:** Qing Wang, Huijing He, Yaoda Hu, Jing Nai, Zhen Song, Hui Pan, Hanze Du, Ji Tu, Zichao Wang, Da Xu, Zhujie Ran, Zhili Chen, Mengwei Zhang, Gang Liu, Guangliang Shan

**Affiliations:** 1 Department of Epidemiology and Statistics, Institute of Basic Medical Sciences & School of Basic Medicine, Chinese Academy of Medical Sciences & Peking Union Medical College, Beijing, China; 2 School of Population Medicine and Public Health, Chinese Academy of Medical Sciences & Peking Union Medical College, Beijing, China; 3 State Key Laboratory of Common Mechanism Research for Major Diseases, Beijing, China; 4 Clinical Laboratory, Beijing Hepingli Hospital, Beijing, China; 5 The Institute of Hematology and Blood Diseases Hospital, Chinese Academy of Medical Sciences and Peking Union Medical College,Tianjin, China; 6 Department of Endocrinology, Peking Union Medical College Hospital, Beijing, China

**Keywords:** Muscle health, Biological age, Sarcopenia, Muscle mass, Grip strength, Chinese adults

## Abstract

To construct biological age (BA)–based normative curves for muscle mass and strength and to determine whether biological age acceleration is associated with impaired muscle health and sarcopenia across adulthood. A total of 29,437 adults aged 20–80 years were analyzed from the China National Health Survey (2023–2024). BA was estimated using the Klemera–Doubal method (KDM) based on clinical biomarkers, and BA acceleration (BAacc) was defined as the residual of BA regressed on chronological age (CA). Appendicular skeletal muscle mass (ASM) and appendicular skeletal muscle mass index (ASMI) were assessed by bioelectrical impedance analysis, while handgrip strength (HGS) and relative handgrip strength (rHGS) were measured by dynamometer. Sex-specific BA-based percentile curves were generated using the Lambda-Mu-Sigma (LMS) method. Logistic regression models evaluated associations of BAacc with low muscle mass (LMM), low muscle strength (LMS), and sarcopenia. BA-based percentile curves revealed steady, sex-specific declines in ASM, ASMI, HGS, and rHGS across BA. Each one-year increase in BAacc was associated with significant reductions in ASMI (β = −0.0064 in men; −0.0041 in women) and rHGS (β = −0.0132 in men; −0.0064 in women) (all p < 0.0001). Accelerated BA (BAacc > 0) was linked to higher odds of LMM (73–77%), LMS (34–37%), and sarcopenia (68–78%), with dose–response relationships across quartiles. Associations were particularly pronounced in adults < 60 years, indicating that biological aging impacts muscle health earlier than the conventional threshold for old age. BA acceleration was strongly associated with impaired muscle mass, strength, and sarcopenia, with stronger effects observed in women and in younger adults. BA-based reference curves demonstrated clear, sex-specific declines in muscle health across adulthood, providing a novel reference framework for future sarcopenia research.

## INTRODUCTION

Sarcopenia, characterized by the progressive loss of skeletal muscle strength, mass, and function, poses a significant public health challenge due to its strong association with adverse outcomes including frailty, falls, fractures, loss of independence, hospitalization, and increased mortality [1]. The global demographic shift towards aging populations, coupled with the rising prevalence of chronic comorbidities (e.g., cardiovascular disease (CVD), chronic kidney disease, diabetes, cancer) that accelerate muscle wasting, substantially amplifies the prevalence and impact of sarcopenia [2, 3]. By 2030, projections indicate that one in five Americans will be aged ≥ 65 years, with those ≥ 85 years representing the fastest-growing segment [4]. Similarly, China faces a rapidly aging society, where the prevalence of sarcopenia notably exceeds 10% in older adults, imposing a considerable socioeconomic burden [5]. Consequently, the prevention and early identification of sarcopenia are paramount for preserving health span, quality of life, and reducing healthcare system strain, underscoring the critical need for effective screening strategies enabling timely interventions.

Current consensus guidelines (e.g., EWGSOP2, AWGS 2019) define and stage sarcopenia based on three core parameters: low muscle strength (typically assessed by handgrip strength (HGS)), low muscle quantity or quality (commonly measured as appendicular skeletal muscle mass (ASM) or appendicular skeletal muscle mass index (ASMI) using Dual-energy X-ray Absorptiometry (DXA) or Bioelectrical Impedance Analysis (BIA)), and low physical performance (e.g., gait speed, chair stand test) [6, 7]. Crucially, these muscle parameters exhibit distinct, often non-linear trajectories across the lifespan. Muscle mass and strength typically peak in early adulthood, plateau, and commence a progressive decline starting around the fourth or fifth decade [8]. This decline accelerates markedly in later decades, significantly influenced by physical inactivity, nutritional status, hormonal changes, and chronic disease burden [9–11]. However, defining “low” levels based solely on chronological age (CA) as a reference frame overlooks substantial inter-individual heterogeneity in biological aging rates among individuals of identical CA. Individuals sharing the same CA may be at vastly different biological stages of muscle health decline.

Biological Age (BA), distinct from CA, quantifies an individual’s functional status and physiological reserve relative to their calendar years. Ideal BA metrics should therefore reflect the multi-system nature of aging [12]. Various approaches to quantify BA have emerged, ranging from single biomarkers to composite algorithms integrating multi-omics data (epigenetic, proteomic, metabolomic) [13–15]. Notably, indices derived from routinely measured clinical parameters have demonstrated superior predictive validity for age-related morbidity and mortality outcomes [14, 16]. Individuals with a BA exceeding their CA are considered biologically “older” and at heightened risk for age-related diseases and functional decline, while those with a lower BA are biologically “younger” and often exhibit greater resilience [17].

This concept is particularly relevant to muscle health, as sarcopenia directly reflects systemic aging processes. Establishing BA-based reference values for muscle mass and strength represents a novel framework for assessing musculoskeletal aging. Rather than referencing muscle metrics to chronological age alone, BAbased curves reflect physiological aging status and capture inter-individual variability. This approach provides a more integrative and personalized basis for evaluating muscle health and may support earlier recognition of individuals at risk for accelerated decline.

Therefore, leveraging a large, nationally representative Chinese cohort, this study aimed to establish, for the first time, BA-based percentile reference curves for muscle mass and strength in adults aged 20–80 years and to evaluate the association between BA acceleration and sarcopenia.

## MATERIALS AND METHODS

### Data source and study population

This study utilized data from the China National Health Survey (CNHS) conducted between 2023 and 2024. The study population comprised community-dwelling adults recruited from five provinces in China: Guangdong, Jiangsu, Liaoning, Jilin, and Tianjin. These provinces were selected to provide broad geographic coverage and socioeconomic diversity. Full details of the CNHS study design and sampling methodology have been published previously [18]. Briefly, a multistage, stratified cluster sampling strategy was employed to ensure representativeness. Participants were recruited from both urban and rural residential areas within the selected provinces, with stratification incorporating local Gross Domestic Product (GDP) levels to reflect socioeconomic gradients. Inclusion criteria were: (1) age ≥ 20 years, and (2) permanent residency in the selected locality for ≥ 5 years. Exclusion criteria were: (1) severe mental disorders (e.g., dementia, schizophrenia) or physical disabilities precluding assessment participation; (2) active-duty military personnel; (3) pregnant or lactating women; and (4) foreign nationals.

From an initial pool of 29,437 eligible individuals identified through sampling, comprehensive data collection was performed. This included: (1) Standardized Interviews: Trained interviewers administered questionnaires capturing sociodemographic characteristics (e.g., age, sex, education, residence, marital status), detailed health behaviors (e.g., physical activity, smoking, alcohol consumption), and comprehensive medical history (including physician-diagnosed chronic diseases and medication use); (2) Physical Measurements: Detailed anthropometric and physical assessments were conducted using standardized protocols and calibrated equipment by trained personnel and (3) Biological Sampling: Following an overnight fast of ≥ 8 hours, fasting venous blood samples were collected from participants. Serum and plasma were separated by centrifugation and aliquoted for storage at -80°C pending biochemical analyses.

The CNHS protocol strictly adhered to the ethical principles outlined in the Declaration of Helsinki. The study received ethical approval from the Bioethical Committee of the Institute of Basic Medical Sciences, Chinese Academy of Medical Sciences (Approval No. 2022177). Written informed consent was obtained from all participants prior to any study procedures.

### Assessment of Muscle Health Parameters

#### Anthropometry

Standing height was measured to the nearest 0.1 cm using a calibrated wall-mounted stadiometer (SECA GmbH, Hamburg, Germany), with participants positioned barefoot in the Frankfurt horizontal plane. Body weight was recorded to the nearest 0.1 kg using a validated multi-frequency segmental bioelectrical impedance analyzer (BIA; Tanita MC-780MA, Tanita Corporation, Tokyo, Japan), with participants wearing light indoor clothing and no footwear.

#### Body Composition and Muscle Mass

The Tanita MC-780MA BIA device, which utilizes multiple frequencies (1 kHz, 5 kHz, 50 kHz, 250 kHz, 500 kHz, 1000 kHz), provided comprehensive body composition analysis. This included quantification of fat mass, lean soft tissue mass, and skeletal muscle mass (SMM) across whole-body, regional (trunk), and appendicular (arms and legs) compartments. The validity of BIA for assessing ASM in our study context is supported by previous research comparing it against DXA [19, 20]. ASM was calculated as the sum of SMM in both arms and legs. ASMI was derived by normalizing ASM to body mass index (BMI; kg/m^2^), where BMI = weight (kg) / height^2^ (m^2^).

#### Muscle Strength

Absolute HGS was assessed using a calibrated Jamar Hydraulic Hand Dynamometer (JAMAR, UK) following standardized protocols [8]. Briefly, after completing a practice test, each participant was asked to squeeze the dynamometer twice as hard as possible for 3 seconds, using the dominant arm, in a standing position with the arms extended straight down to the side. The highest value recorded from either hand was used for analysis. Relative Handgrip Strength (rHGS) was calculated as absolute HGS (kg) divided by BMI.

#### Assessment of BA

To calculate BA, the Klemera–Doubal method (KDM) was applied using selected clinical biomarkers (Supplementary Tables). Initially, 19 candidate biomarkers were considered, and those with a Pearson correlation coefficient greater than 0.10 with chronological age (CA) in the reference population were retained. As a result, 12 biomarkers for men and 13 biomarkers for women were included in the KDM algorithm. Regression analyses between each biomarker and CA were conducted to estimate KDM values [21]. Biological aging was then defined by comparing KDM with CA: individuals with KDM greater than their CA were classified as experiencing accelerated aging, whereas those with KDM lower than their CA were considered to have decelerated aging [22].

#### Lifestyle Factors

Data on key lifestyle factors, including cigarette smoking status (current, former, never), alcohol consumption (current, former, never), and physical activity levels, were collected using standardized, interviewer-administered questionnaires. Detailed definitions and assessment methods for these variables are consistent with those employed in CNHS and have been published previously [18].

#### Chronic disease definitions

Hypertension as systolic blood pressure (SBP) ≥ 140 mmHg and/or diastolic blood pressure (DBP) ≥ 90 mmHg (measured using a calibrated automated oscillometric device [e.g., Omron HEM-7136, Omron Healthcare, Kyoto, Japan] after ≥ 5 minutes of rest, averaged three readings), a self-reported physician diagnosis of hypertension, or current use of antihypertensive medication. Diabetes was defined as fasting plasma glucose (FPG) ≥ 7.0 mmol/L (measured from venous blood samples using standardized enzymatic methods), a self-reported physician diagnosis of diabetes, or current use of glucose-lowering medication (oral agents or insulin). Other Chronic Conditions: History of CVDs (e.g., myocardial infarction, stroke, heart failure), musculoskeletal disorders (e.g., osteoarthritis, rheumatoid arthritis, gout), neurological disorders (e.g., Parkinson’s disease, stroke sequelae), and cancers were ascertained based on self-reported physician diagnosis.

### Statistical analysis

We analyzed data from two overlapping groups of Chinese adults. Analysis 1 (n = 29,437) included all participants with complete baseline data for clinical biomarkers, physical function, and body composition, and was used to examine the associations of BA with LMM, LMS, and sarcopenia. Analysis 2 (n = 18,367) comprised a healthy reference sample, excluding individuals with cardiovascular, metabolic, musculoskeletal, neurological diseases, or cancer, and was used to generate BA–specific percentile curves for muscle health parameters.

Descriptive statistics were used to summarize the distributions of muscle health indicators. Sex- and BA-specific percentiles (1^st^, 5^th^, 25^th^, 50^th^, 75^th^, 95^th^, and 99^th^) for HGS, rHGS, ASM, and ASMI were estimated using the Lambda-Mu-Sigma (LMS) method, which models the median (μ), coefficient of variation (σ), and skewness (λ) [23]. Percentile curves were fitted using the gamlss package in R (version 4.4.3) with cubic smoothing splines.

Logistic regression models were used to assess the associations between BA and the risk of (1) LMM (defined as ASM below the sex- and 5-year age group–specific 20^th^ percentile), (2) LMS (defined as HGS below the sex- and 5-year age group–specific 20^th^ percentile), and (3) sarcopenia (defined as concurrent low ASM and HGS). Percentile thresholds were derived separately for men and women within 5-year chronological age groups (e.g., 20–24, 25–29, etc.). All sex-stratified models were adjusted for age, residence, education, marital status, smoking, alcohol use, physical activity, hypertension, and diabetes. A series of sensitivity analyses were conducted. First, we adjusted HGS and ASMI for body weight. Second, we stratified participants into a relatively healthy subset (n = 18,367) and a higher-risk subset (n = 11,070) to assess consistency across risk profiles. Third, we pooled males and females and repeated the analyses to evaluate sex-combined associations. Fourth, we stratified by age (< 60 vs. ≥ 60 years) to examine potential heterogeneity across life stages. Fifth, we compared unadjusted, partially adjusted, and fully adjusted models to test the impact of covariate adjustment levels. Sixth, we assessed the sensitivity of results to different biomarker sets and parameter sources used in constructing KDM, including the application of NHANES-trained parameters to the Chinese biomarker set and the combined use of NHANES biomarkers and parameters. Seventh, to address potential residual confounding by socioeconomic status (SES), we constructed composite SES classes using latent class analysis (LCA). Four observed SES indicators—educational attainment, household income, medical insurance coverage, and occupational category—were harmonized into ordered levels. Educational attainment, income, and medical insurance coverage were categorized into three levels (low, medium, and high), while occupational category was classified into four levels (low, medium-low, medium-high, and high). A series of LCA models were fitted, and both 3-class (low, medium, high) and 4-class (low, medium-low, medium-high, high) solutions were retained based on model fit indices, including Akaike information criterion (AIC), Bayesian information criterion (BIC), entropy, parsimony, and substantive interpretability. Class membership was assigned according to the maximum posterior probability. We then (1) adjusted for the LCA-derived SES classes as covariates in the regression models and (2) conducted SES-stratified analyses under both the 3-class and 4-class solutions to examine the robustness of the associations and assess potential effect modification by SES. Eighth, to further account for the potential influence of dietary habits, we constructed a dietary quality score based on eight commonly consumed food groups: vegetables (≥ 5 days/week), fruits (≥ 5 days/week), milk (≥ 3 days/week), soy products (≥ 3 days/week), seafood (≥ 1–2 days/week), eggs (≥ 3 days/week), meat (1–4 days/week), and preserved foods (≤ 3 times/month). For each component, participants received 1 point if the recommended intake frequency was met and 0 otherwise, yielding a total score ranging from 0 to 8. The dietary score was included as an additional covariate in the regression models. Lastly, to adjust for potential confounding by pharmacological treatment, we additionally accounted for medication use, including antihypertensive, antidiabetic, lipid-lowering, and urate-lowering drugs. Participants were classified as having a medication history if they reported current or past use of any of these medications; otherwise, they were categorized as having no medication history. This medication-use variable was included as an additional covariate in the fully adjusted models.

A flow diagram of the study design is provided in Figure S1. All statistical analyses were performed using R version 4.4.3 (R Foundation for Statistical Computing, Vienna, Austria). A two-sided p-value < 0.05 was considered statistically significant.

### External prospective validation in the UK Biobank

To address concerns regarding causal inference and potential reverse causation, we conducted an external prospective validation in the UK Biobank, a large population-based cohort with longitudinal follow-up. The UK Biobank was selected for validation due to its independent recruitment, standardized phenotyping, and availability of repeated assessments of muscle strength and body composition.

The validation sample included 31,429 participants aged 40–70 years who were free LMM, LMS and sarcopenia at baseline. Participants were followed for a median of 9.7 years, during which incident LMM, LMS and sarcopenia were ascertained based on follow-up measurements of appendicular skeletal muscle index and handgrip strength. Cox proportional hazards models were used to examine the association between baseline biological age acceleration and subsequent risk of incident LMM and LMS.

## RESULTS

### Participants’ characteristics

Baseline characteristics are summarized in Table 1. The mean age of participants was 53.9 ± 12.9 years; 36.5% were men and 60.5% were urban residents. The prevalence of overweight and obesity was 55.6%, hypertension 39.8%, and diabetes 12.1%. Sarcopenia was present in 9.1% of participants.

**TABLE 1:** Baseline Characteristics^a^

Characteristic	Analysis 1 (N=29437)	Analysis 2 (N=18367)
**Age (years)**	53.94 (12.91)	50.81 (12.88)

**Body mass index (BMI, kg/m^2^)**		

Normal weight (18.5 to < 24)	12274 (41.7%)	7915 (43.1%)
Obese (≥ 28)	5011 (17.0%)	3029 (16.5%)
Overweight (24 to < 28)	11370 (38.6%)	6890 (37.5%)
Underweight (< 18.5)	782 (2.7%)	533 (2.9%)

**Sex (male)**	10735 (36.5%)	6732 (36.7%)

**Ethnicity (Han)**	23621 (80.2%)	14718 (80.1%)

**Smoking status**		

Never smoker	22128 (75.2%)	14085 (76.7%)
Former smoker	2093 (7.1%)	990 (5.4%)
Current smoker	5206 (17.7%)	3283 (17.9%)

**Drinking status**		

Never drinker	20911 (71.1%)	13145 (71.6%)
Former drinker	1219 (4.1%)	501 (2.7%)
Current drinker	7292 (24.8%)	4707 (25.6%)

**Residence (Urban)**	17796 (60.5%)	11216 (61.1%)

**Education level**		

College or above	8044 (27.4%)	5850 (31.9%)
High school or equivalent	6222 (21.2%)	3629 (19.8%)
Less than high school	15100 (51.4%)	8848 (48.3%)

**Marital status (Currently married)**	25528 (86.7%)	15928 (86.7%)

**Major diseases** ^b^		

Hypertension	11703 (39.8%)	6239 (34.0%)
Diabetes	3569 (12.1%)	1789 (9.7%)

**Components of KDM**		

Albumin, g/L	46.00 (44.30–47.60)	46.10 (44.50–47.70)
Alkaline phosphatase, U/L	80.00 (65.00–97.00)	77.00 (62.00–94.00)
Alanine aminotransferase, U/L	17.00 (13.00–24.00)	17.00 (13.00–24.00)
Aspartate aminotransferase, U/L	22.00 (19.00–27.00)	22.00 (18.00–26.00)
Total cholesterol, mmol/L	5.35 (4.68–6.07)	5.30 (4.64–6.02)
Creatinine, umol/L	58.00 (52.00–64.00)	58.00 (52.00–64.00)
Fasting glucose, mmol/L	5.36 (5.03–5.84)	5.32 (5.00–5.76)
Gamma-glutamyl transferase, U/L	22.00 (16.00–34.00)	22.00 (16.00–33.00)
HDL cholesterol, mmol/L	1.20 (1.02–1.44)	1.20 (1.02–1.43)
LDL cholesterol, mmol/L	3.08 (2.53–3.68)	3.04 (2.52–3.63)
Lymphocyte count, × 10^9^/L	2.07 (1.70–2.51)	2.10 (1.73–2.53)
Platelet count, × 10^9^/L	244.00 (206.00–285.00)	248.00 (210.00–289.00)
Red blood cell count, × 10¹^2^/L	5.10 (4.81–5.38)	5.14 (4.85–5.42)
Total triglyceride, mmol/L	1.40 (1.00–2.00)	1.30 (0.90–1.90)
Uric acid, umol/L	389.00 (332.00–453.00)	395.00 (337.00–458.00)
Urea, mmol/L	5.10 (4.30–6.10)	5.00 (4.20–6.00)
Systolic BP, mmHg	125.00 (114.00–139.00)	124.00 (112.00–137.00)
Diastolic BP, mmHg	75.00 (68.00–83.00)	75.00 (68.00–82.00)

**Skeletal Muscle and Bone Status**		

Low muscle mass (LMM)	5898 (20.0%)	3755 (20.4%)
Low muscle strength (LMS)	5898 (20.0%)	3539 (19.3%)
Sarcopenia	2681 (9.1%)	1682 (9.2%)

a full dataset comprising the entire study population (n = 29,437) for Analysis 1, and a reference dataset of apparently healthy individuals (n = 18,367) for Analysis 2, excluding those with CVD, cancer, musculoskeletal, or neurological disorders.

b Hypertension was defined as SBP ≥ 140 mmHg and/or DBP ≥ 90 mmHg (measured after ≥ 5 minutes of rest using a calibrated automated device), self-reported physician diagnosis, or current use of antihypertensive medication. Diabetes was defined as fasting plasma glucose (FPG) ≥ 7.0 mmol/L, self-reported diagnosis, or use of glucose-lowering medication (oral or insulin).

#### Characteristics of KDM-BA

KDM-BA ranged from 11.0 to 89.4 years, with a mean of 53.9 years and a median of 56.1 years (SD = 13.5). Figure S2 presents the distributions of KDM-BA, KDM-BA acceleration (KDM-BAacc). As expected, KDM-BA was highly correlated with chronological age (CA) [21].

### The sex-specific values of ASM/ASMI and HGS/rHGS

**FIG. 1:**
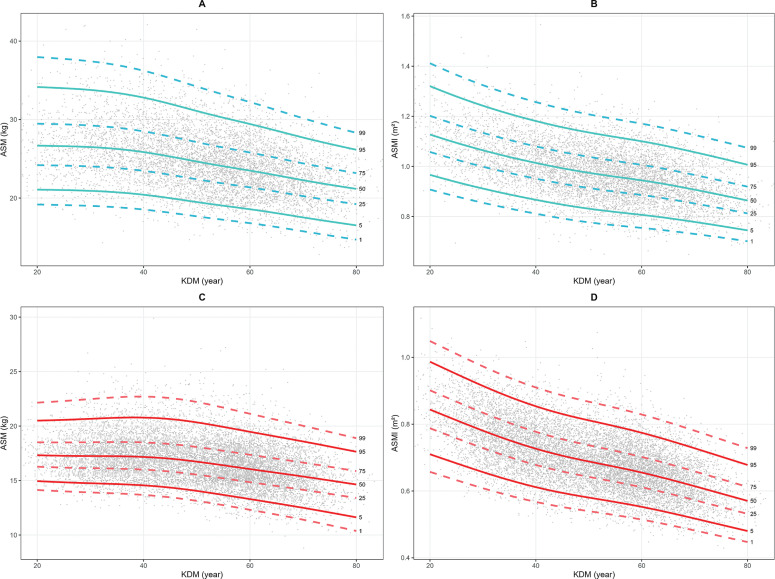
Appendicular skeletal muscle mass and appendicular skeletal muscle mass index reference percentiles by BA for Chinese adults aged 20–80 years, stratified by sex The solid lines represent the 5^th^, 50^th^, and 95^th^ percentiles, and the dotted lines represent the 1^st^, 25^th^, 75^th^, and 99^th^ percentiles, plotted in ascending order from bottom to top.(A) ASM in men;(B) ASMI in men; (C) ASM in women;(D) ASMI in women.

In general, men exhibited higher ASM and ASMI values than women throughout the BA spectrum (Table S3). In men, median ASM values declined from approximately 26.2 kg at BA 20 years to 21.4 kg at BA 80 years (Figure 1A). This suggests a downward shift in muscle mass with biological aging. ASMI in men (Figure 1B) showed a similar declining trend, with median values decreasing from 1.10 to approximately 0.85. Women had lower absolute values of ASM and ASMI compared to men. Median ASM declined from 17.3 kg to 14.5 kg (Figure 1C), while ASMI decreased from 0.82 to 0.58 (Figure 1D) between BA 20 and 80 years. The sex gap was most pronounced in early adulthood and narrowed slightly in later BA decades. The percentile trajectories also revealed that the decline in ASMI was more linear and consistent across age than that of raw ASM, which showed mild preservation during midlife followed by a steeper drop in older adults.

**FIG. 2:**
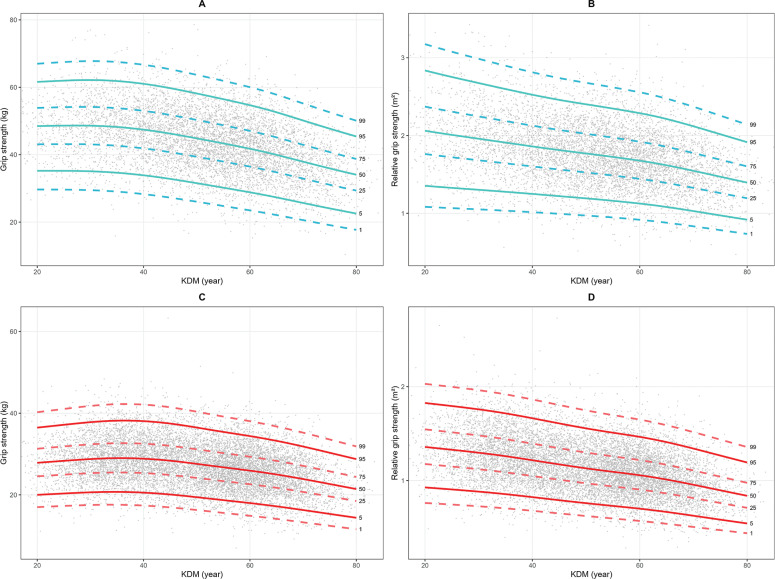
Absolute and relative hand grip strength reference percentiles by BA for Chinese adults aged 20–80 years, stratified by sex The solid lines represent the 5^th^, 50^th^, and 95^th^ percentiles, and the dotted lines represent the 1^st^, 25^th^, 75^th^, and 99^th^ percentiles, plotted in ascending order from bottom to top.(A) Absolute hand grip strength in men; (B) Relative hand grip strength in men;(C) Absolute hand grip strength in women;(D) Relative hand grip strength in women.

Similar sex-related differences were observed for HGS. Men consistently demonstrated higher HGS than women across all BA groups (Table S4). In men (Figure 2A), median HGS declined from around 47.6 kg at BA 20 years to approximately 35.2 kg at BA 80 years. In contrast, women (Figure 2C) had lower absolute values, with the median HGS decreasing from 27.8 kg to about 21.4 kg across the same age range. rHGS showed smoother trajectories with a more linear decline. In men (Figure 2B), rHGS decreased from a median of 2.02 to around 1.40 between BA 20 and 80 years, while in women (Figure 2D), the decline was from 1.35 to approximately 0.88. These sex-specific trends illustrate that both absolute and relative muscle strength decline with increasing BA, with sharper decreases observed in older adults.

### Association of BA with muscle outcomes

As shown in Table 2, higher KDM-BAacc was significantly associated with lower ASMI and reduced rHGS in both men and women. Modeled continuously, each one-year increase in KDM-BAacc was associated with a 0.0064-unit and 0.0132-unit decline in ASMI and rHGS, respectively, in men, and a 0.0041-unit and 0.0064-unit decline, respectively, in women (all p < 0.0001). In parallel, individuals with KDM-BAacc had 73–77% higher odds of LMM, 34–37% higher odds of LMS, and 68–78% higher odds of sarcopenia compared with those with non-accelerated aging. In quartile-based models, a clear dose–response pattern emerged. Compared with individuals in the lowest quartile (Q1) of KDM-BAacc, those in Q4 had markedly increased risks of LMM (OR = 2.37 in men; 2.82 in women), LMS (OR = 1.76 in men; 1.60 in women), and sarcopenia (OR = 2.62 in men; 2.50 in women) (all p < 0.0001).

**TABLE 2:** Associations between KDM-BAacc and muscle-related indicators (fully adjusted model)^a^

Biological age	ASMI					Relative Grip Strength			

	Coefficients (SE)		p-value			Coefficients (SE)		p-value	
KDM-BAacc (Male)^b^	−0.0064 (0.0002)		< 0.0001			−0.0132 (0.0010)		< 0.0001	

**KDM-BAacc (Quartile)**									
Q1	Ref					Ref			
Q2	−0.0280 (0.0025)		< 0.0001			−0.0645 (0.0098)		< 0.0001	
Q3	−0.0432 (0.0025)		< 0.0001			−0.0825 (0.0099)		< 0.0001	
Q4	−0.0607 (0.0025)		< 0.0001			−0.1401 (0.0099)		< 0.0001	
KDM-BAacc (Female)	−0.0041 (0.0001)		< 0.0001			−0.0064 (0.0005)		< 0.0001	

**KDM-BAacc (Quartile)**									
Q1	Ref					Ref			
Q2	−0.0151 (0.0014)		< 0.0001			−0.0144 (0.0049)		0.0035	
Q3	−0.0251 (0.0015)		< 0.0001			−0.0330 (0.0050)		< 0.0001	
Q4	−0.0419 (0.0015)		< 0.0001			−0.0630 (0.0053)		< 0.0001	


**Biological age**	**LMM**			**LMS**			**Sarcopenia**		

	**N_case/N_ total**	**Odds ratio (95% CI)**	**p-value**	**N_case/N_ Odds ratio (95% total CI)**	**p-value**	**N_case/N_ total**	**Odds ratio (95% CI)**	**p-value**

KDM-BAacc (Male)^b^	2152/10735	1.73 (1.56–1.91)	< 0.0001	2152/10735 1.34 (1.21–1.48)	< 0.0001	892/10735	1.68 (1.46–1.94)	< 0.0001

**KDM-BAacc (Quartile)**									
Q1	383/2684	Ref		444/2684 Ref		142/2684	Ref	
Q2	484/2684	1.4 (1.21–1.63)	< 0.0001	537/2684 1.35 (1.17–1.56)	< 0.0001	212/2684	1.65 (1.32–2.06)	< 0.0001
Q3	572/2684	1.79 (1.55–2.07)	< 0.0001	533/2684 1.39 (1.21–1.61)	< 0.0001	227/2684	1.84 (1.48–2.3)	< 0.0001
Q4	713/2683	2.37 (2.06–2.74)	< 0.0001	638/2683 1.76 (1.53–2.03)	< 0.0001	311/2683	2.62 (2.12–3.25)	< 0.0001
KDM-BAacc (Female)	3746/18702	1.77 (1.63–1.92)	< 0.0001	3746/18702 1.37 (1.26–1.48)	< 0.0001	1789/18702	1.78 (1.59–1.99)	< 0.0001

**KDM-BAacc (Quartile)**									
Q1	523/4676	Ref		707/4676 Ref		245/4676	Ref	
Q2	802/4676	1.54 (1.36–1.74)	< 0.0001	815/4676 1.11 (0.99–1.24)	0.0748	338/4676	1.27 (1.07–1.51)	0.0071
Q3	977/4675	1.83 (1.62–2.06)	< 0.0001	969/4675 1.30 (1.16–1.45)	< 0.0001	459/4675	1.64 (1.39–1.94)	< 0.0001
Q4	1444/4675	2.82 (2.5–3.18)	< 0.0001	1255/4675 1.60 (1.43–1.8)	< 0.0001	747/4675	2.50 (2.13–2.95)	< 0.0001

a fully adjusted for age, socioeconomic status (residence, education level, marital status), lifestyle behaviors (smoking, drinking, physical activity), and clinical comorbidities (hypertension and diabetes)

b For ASMI and relative grip strength, KDM-BAacc was modeled as a continuous variable (per 1-year increase) in linear regression models. For LMM, LMS, and sarcopenia, KDM-BAacc was dichotomized (accelerated vs. non-accelerated aging) in logistic regression models, with all outcomes treated as binary variables.

**FIG. 3:**
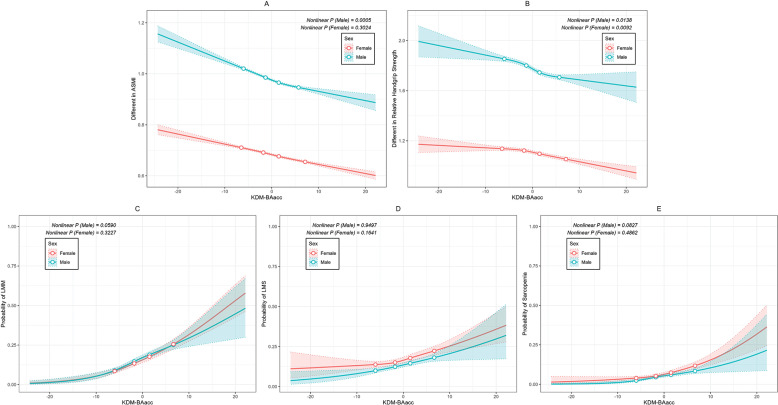
Sex-specific nonlinear associations between KDM-BAacc and muscle-related outcomes A: ASMI;B: Relative Grip Strength;C: LMM;D: LMS; E: Sarcopenia.Solid line: Point estimation; Dash line: Confidence limits; Dots: Knots (5^th^, 35^th^, 65^th^, and 95^th^ percentiles). Restricted cubic spline regression model adjusted for age, socioeconomic status (residence, education level, marital status), lifestyle behaviors (smoking, drinking, physical activity), and clinical comorbidities (hypertension and diabetes).

Figure 3 illustrates the nonlinear associations between KDMBAacc and various muscle-related outcomes stratified by sex. For ASMI and rHGS (Figure 3A–B), higher KDM-BAacc was associated with a significant decline in ASMI and rHGS (Figure 3A–B). Nonlinear associations were observed for all outcomes except ASMI in women, with stronger evidence of nonlinearity in men. Notably, the decline in HGS associated with each unit increase in KDM-BAacc was steeper in men than in women, which may be explained by men’s higher baseline muscle strength and the consequently more pronounced decline during aging. For LMM, LMS and sarcopenia (Figure 3C–E), a dose–response increase in the predicted probability of LMM, LMS, and sarcopenia was observed with advancing KDMBAacc. These patterns were generally monotonic and similar between sexes, though the slope of risk increase was slightly steeper in women, particularly for sarcopenia. However, tests for nonlinearity were not statistically significant for most outcomes (all nonlinear P > 0.05).

### Sensitivity analyses

To ensure the robustness of our main findings, we conducted a series of sensitivity analyses. First, we adjusted HGS and ASMI for body weight (Table S5). Second, we conducted risk stratification analyses by dividing the population into two subgroups: a relatively healthy subset (N = 18,367), which was used to construct reference curves, and a remaining higher-risk population (N = 11,070)(Table S6, Table S7). Third, we combined males and females into a pooled dataset and repeated the analyses. The results remained consistent with those observed in sex-stratified models (Table S8). Fourth,we stratified the population by age (< 60 vs. ≥ 60 years) to examine potential heterogeneity across life stages (Table S9, Table S10). The associations between BAacc and muscle-related outcomes remained significant in both age groups. Fifth, we examined the impact of different covariate adjustment levels by comparing unadjusted, partially adjusted (Model 1 and Model 2), and fully adjusted models (Table S11). The associations remained stable and statistically significant across all models, suggesting that the results were not driven by confounding due to these lifestyle or demographic factors. Across these five analyses, the associations between BA acceleration and muscle-related outcomes remained consistent, supporting the robustness of the main results. Sixth, we evaluated the sensitivity of our findings to different biomarker sets and parameter sources used in constructing KDM. When applying NHANES-trained parameters to the Chinese biomarker set, the associations between KDM-BA and sarcopenia-related outcomes largely remained significant among females, while becoming non-significant for some outcomes among males (Table S12). When using both the NHANES biomarker set and NHANES-trained parameters, the associations were attenuated in both sexes, with most outcomes remaining significant among females but only partially retained among males (Table S13). These findings underscore the importance of context-specific biomarker selection and parameter calibration in BA modeling across populations.

In addition, to further address potential residual confounding by socioeconomic status, dietary status, and pharmaceutical usage, we performed additional sensitivity analyses (Tables S14–S19). After further adjustment for LCA-derived SES classes using either the 3-class (SES3) or 4-class (SES4) solutions, the associations between KDM-BAacc and muscle-related indicators remained materially unchanged in both men and women (Tables S14 and S15). We further conducted SES-stratified analyses. The associations between KDMBAacc and LMM, LMS, and sarcopenia were consistently observed across all SES strata under both the SES3 and SES4 classifications (Tables S16 and S17), indicating that the observed relationships were not driven by socioeconomic confounding. Moreover, additional adjustment for dietary status using the dietary score (Table S18) and for pharmaceutical usage (antihypertensive, antidiabetic, lipidlowering, and urate-lowering medications; Table S19) yielded results that were highly consistent with the main findings, with no meaningful attenuation of effect estimates. Collectively, these analyses provide further support for the robustness of the associations between KDM-based biological age acceleration and impaired muscle health outcomes.

### Exploratory analyses

In exploratory analyses, we examined potential determinants of KDMBAacc and adverse muscle-related outcomes using sex-stratified multivariable logistic regression models (Table S20). Healthier dietary patterns were consistently associated with lower odds of LMM, LMS and sarcopenia in both men and women, showing clear dose–response relationships, while higher educational attainment and socioeconomic status were generally associated with more favorable muscle health, particularly among women. Moderate-to-high physical activity frequency was consistently associated with lower odds of adverse muscle outcomes; however, its associations with KDM-BAacc, especially among men, were less consistent. This pattern likely reflects reverse causation, whereby individuals with accelerated biological aging may reduce or modify physical activity due to declining physiological reserve, in contrast to muscle mass and strength, which represent more stable structural and functional outcomes influenced by long-term activity exposure. Among women, inverse associations observed for smoking should be interpreted with caution given the very low prevalence of current and former smokers, which may have resulted in limited statistical stability. Overall, these findings are descriptive and hypothesis-generating, and causal inferences cannot be drawn due to the cross-sectional design.

### External prospective validation of BA–muscle associations in the UK Biobank

To address concerns regarding causal inference and potential reverse causation, we conducted a prospective external validation in the UK Biobank. After excluding participants with prevalent LMM, LMS and sarcopenia at baseline, 31,429 individuals (15,216 men and 16,213 women) were followed for a median of 9.7 years. During follow-up, 2,608 men and 1,786 women developed incident LMM, 2,562 men and 2,404 women developed incident LMS, and 820 men and 706 women developed incident sarcopenia. In fully adjusted Cox proportional hazards models, higher baseline KDM BAacc was consistently associated with an increased risk of all muscle-related outcomes in both sexes (Table 3).These prospective findings support a temporal association between accelerated biological aging and subsequent impairment in muscle health.

**TABLE 3:** Association between accelerated KDM-BAacc and incident muscle-related outcomesin the UK Biobank (Cox models,fully adjusted model)^a^

Biological age	LMM			LMS			Sarcopenia		

Biological age	N_case/N_ total	Hazard ratio (95% CI)	p-value	N_case/N_ total	Hazard ratio (95% CI)	p-value	N_case/N_ total	Hazard ratio (95% CI)	p-value
**Male**									

KDM-BAacc (Continuous)	2608/15216	1.08 (1.06–1.09)	< 0.0001	2562/15216	1.06 (1.05–1.07)	< 0.0001	820/15216	1.12 (1.09–1.14)	< 0.0001

KDM-BAacc (Binary)	2608/15216	1.52 (1.4–1.65)	< 0.0001	2562/15216	1.34 (1.23–1.45)	< 0.0001	820/15216	1.97 (1.71–2.28)	< 0.0001

**Female**									

KDM-BAacc (Continuous)	1786/16213	1.16 (1.14–1.19)	< 0.0001	2404/16213	1.07 (1.06–1.09)	< 0.0001	706/16213	1.17 (1.14–1.21)	< 0.0001

KDM-BAacc (Binary)	1786/16213	1.77 (1.6–1.95)	< 0.0001	2404/16213	1.32 (1.21–1.43)	< 0.0001	706/16213	1.85 (1.58–2.16)	< 0.0001

a fully adjusted for age, ethnicity, socioeconomic status (Townsend deprivation index), lifestyle behaviors (smoking, drinking, and physical activity), and clinical comorbidities (hypertension and diabetes).

^b^ Linear regression models were used for continuous outcomes, and multivariable logistic regression models were used for binary outcomes.

## DISCUSSION

In this large, population-based study of Chinese adults aged 20–80 years, we constructed, for the first time, BA-based reference curves for HGS, rHGS, ASM, and ASMI, and revealed sex-specific patterns of age-related declines in muscle health, with women showing generally steeper declines than men. Further analyses demonstrated that BAacc was significantly associated with LMS, LMM, and sarcopenia. In age-stratified analyses, the associations were more pronounced among participants younger than 60 years.

To date, no previous study has established biological age–based normative reference distributions for skeletal muscle mass or strength. Our study therefore introduces a new reference framework by anchoring muscle aging trajectories to biological rather than chronological time. Nevertheless, prior evidence indicates a close link between biological aging and musculoskeletal decline. Accelerated biological aging has been observed in skeletal muscle tissue, as evidenced by epigenetic age acceleration and enrichment of senescence-related transcriptional pathways [24], and population-based studies using DNA methylation clocks have demonstrated robust inverse associations between biological age acceleration and grip strength [25]. In addition, grip strength has been widely recognized as a biomarker of aging, with strong cross-sectional and prospective associations with multimorbidity, disability, hospitalization, and mortality [26]. Our findings are consistent with this body of evidence, demonstrating that accelerated biological aging is associated with both reduced muscle mass and impaired muscle strength at the population level. Although different biological age algorithms were applied across studies—including epigenetic clocks and clinical biomarker–based models—the direction of association is remarkably concordant, supporting the robustness and biological plausibility of linking accelerated aging processes to musculoskeletal decline.

Consistent with previous studies using clinical biomarkers or epigenetic clocks, which have shown BA to be a robust predictor of morbidity, mortality, and functional decline [27, 28], our findings further support the utility of BA in musculoskeletal aging research. For example, Belsky et al. [27] demonstrated that BA estimates in young and middle-aged adults predicted physical and cognitive decline over the subsequent decades, supporting the view that BA captures early subclinical aging processes. However, few studies have examined these relationships in Asian populations, particularly in a large, nationally representative cohort. The Japanese study by Ishizaki et al. [29] linked higher “BA” scores based on metabolic and inflammatory markers to poorer physical performance, but their sample was limited to older adults aged ≥ 65 years. Our study extends these findings to a broader age range and shows that BA-associated declines can be detected decades earlier, with stronger associations observed in adults younger than 60 years, particularly for sarcopenia, thereby highlighting midlife as a critical window for intervention.

The sex-specific differences we observed—where associations were more consistent and statistically robust in women—are partly consistent with earlier work. For instance, Yu et al. [30, 31] reported stronger associations between BA and frailty in older Chinese women than in men, potentially due to differences in fat-to-muscle ratio trajectories, hormonal decline patterns, and lifestyle exposures. Conversely, some Western studies have reported different sex-specific patterns. For example, Phyo et al. found that in older Australians, several aging-related biomarkers, including DNA methylation age acceleration, were more strongly associated with certain health outcomes such as chronic kidney disease in men, whereas associations with obesity and depression were stronger in women [32]. These variations emphasize that sex differences may differ by ethnicity, body composition norms, and environmental or sociocultural contexts, reinforcing the need for tailored reference standards.

Several biological mechanisms may explain the observed associations between BAacc and sarcopenia-related outcomes. First, BAacc reflects cumulative multisystem dysregulation, including endocrine, inflammatory, metabolic, and cardiovascular pathways, which collectively influence muscle protein synthesis and degradation [33]. Chronic low-grade inflammation, characterized by elevated interleukin-6 (IL-6) and tumor necrosis factor-α (TNF-α), has been shown to impair muscle anabolism and promote proteolysis [34], while also contributing to accelerated biological aging [35]. Second, oxidative stress and mitochondrial dysfunction are central features of both accelerated BA and sarcopenia. Mitochondrial DNA damage and reduced oxidative phosphorylation capacity compromise muscle energetics, leading to fatigue and muscle fiber atrophy [36]. These changes are mirrored in BA algorithms through biomarkers such as albumin, creatinine, and inflammatory proteins, suggesting that BA may act as an integrative marker of mitochondrial health. Third, hormonal dysregulation—including declines in testosterone, estrogen, and growth hormone—plays a pivotal role in muscle and bone loss with aging. Estrogen deficiency after menopause accelerates both osteoporosis and sarcopenia via effects on muscle satellite cell function, calcium homeostasis, and collagen cross-linking [37–41]. Lastly, neurological factors may contribute: aging is associated with loss of motor units, reduced neuromuscular junction integrity, and slower nerve conduction velocity [42]. As BAacc captures cumulative physiological wear, it may indirectly reflect these neurogenic components of sarcopenia.

This study has several notable strengths. First, it leveraged a large, geographically diverse Chinese cohort, enhancing the generalizability of the findings. Second, BA was estimated using a validated KDM algorithm based on routinely measured clinical biomarkers, ensuring methodological robustness. Third, a wide range of sensitivity analyses—including age-stratified, disease-stratified, and biomarker set–specific models—demonstrated the robustness of our results. Several limitations should also be acknowledged. First, although the primary analyses were based on a cross-sectional Chinese cohort and therefore cannot establish causality on their own, we have added an external prospective validation in the UK Biobank. These findings support the temporal direction of the association and substantially reduce the likelihood that our main results are explained solely by reverse causation. Nevertheless, because the UK Biobank population differs from the Chinese cohort in ethnicity, body composition profiles, and lifestyle patterns, future longitudinal studies in Chinese populations are warranted to further confirm these temporal associations. Second, reliance on bioelectrical impedance analysis (BIA) rather than dual-energy X-ray absorptiometry (DXA) for the assessment of muscle mass may introduce systematic measurement error related to hydration status, adiposity distribution, and metabolic conditions. Although DXA is the reference standard for body composition assessment, its routine use is often not feasible in largescale, field-based epidemiological surveys due to logistical constraints, cost, throughput requirements, and radiation exposure. Therefore, BIA is commonly adopted as a pragmatic and scalable alternative in population-based studies. Importantly, previous validation studies in Asian populations have consistently shown that BIA tends to overestimate appendicular skeletal muscle mass compared with DXA [19]. Such systematic overestimation would lead to under-detection of low muscle mass and sarcopenia, resulting in non-differential misclassification that would be expected to bias observed associations toward the null. Consequently, the true associations between biological age acceleration and impaired muscle health may be stronger than those observed in the present study. In addition, we observed highly consistent associations between biological age acceleration and muscle-related outcomes in an external validation analysis using the UK Biobank cohort, in which body composition was assessed using imaging-based methods. The concordance of findings across different cohorts and measurement platforms suggests that our conclusions are unlikely to be driven solely by systematic measurement bias associated with BIA. Third, while the KDM algorithm is well established, several biomarkers included in the algorithm (e.g., albumin, creatinine, and inflammatory markers) are biologically related to muscle metabolism and function. This may introduce partial construct overlap between biological age and muscle health and could contribute to inflation of statistical associations due to potential circularity. We therefore interpret biological age as an integrative, multisystem aging signal rather than a muscle-specific biological construct. To address this concern, we conducted sensitivity analyses using alternative biomarker sets and parameter sources for KDM construction. Across these models, the direction of the associations between biological age acceleration and muscle-related outcomes remained consistent, although effect sizes were attenuated in some specifications, particularly among men. These findings suggest that while construct overlap may influence the magnitude of effect estimates, the overall association is robust. Fourth, although we conducted extensive sensitivity analyses with additional adjustment for dietary status, pharmaceutical usage, and a latent class–derived socioeconomic status indicator, residual confounding inherent to observational studies cannot be completely excluded. In particular, detailed assessments of mental health, psychosocial stress, specific physical activity patterns (e.g., resistance training and sedentary behavior), inflammatory cytokine profiles, and medication dosage and duration were not available in the current survey and therefore could not be explicitly modeled. Future longitudinal studies incorporating comprehensive psychosocial, behavioral, and biological measures are warranted to further elucidate these relationships.

The BA-based curves established in this study provide reference data that may help characterize age-related changes in muscle health across adulthood. Future longitudinal studies are warranted to explore how changes in biological aging relate to trajectories of muscle decline and functional outcomes.

## CONCLUSIONS

This large, population-based study established, for the first time, BA-based reference curves for muscle mass and strength in a Chinese cohort. We found that BA acceleration was strongly associated with low muscle mass, low muscle strength, and sarcopenia, with associations more pronounced in women and in adults younger than 60 years.

## Supplementary Material

Accelerated biological aging and risk of sarcopenia: evidence from 29,000 Chinese adults

## Data Availability

The CNHS data that support the findings of this study are available on reasonable request from the corresponding author. We are grateful to all participants and investigators of the UK Biobank. This research has been conducted using the UK Biobank resource (https://www.ukbiobank.ac.uk) under application number 783705.

## Clinical trial registration

Clinical trial number: not applicable.
