# Supplementary material for: Accelerated biological aging and risk of sarcopenia: evidence from 29,000 Chinese adults
Source: Biol Sport. 2026 Apr 13;43:1097–109. doi: 10.5114/biolsport.2026.159572 (PMC13343269; doi:10.5114/biolsport.2026.159572)
Supplement: Accelerated biological aging and risk of sarcopenia: evidence from 29,000 Chinese adults [file JBS-43-57611-s1.pdf]

SUPPLEMENTARY MATERIALS

SUPPLEMENTARY METHODS DESCRIPTION

Calculation of Biological Age (BA) using the Klemera-Doubal method (KDM-BA) and Biological Age Acceleration (KDM-AA) derived from the same approach

$$\mathrm{KDM-BA} = \frac{\sum_{j=1}^{m}(x_j - q_j)\left(\frac{k_j}{s_j^2}\right) + \frac{CA}{s_{BA}^2}}{\sum_{j=1}^{m}\left(\frac{k_j}{s_j^2}\right) + \frac{1}{s_{BA}^2}} \quad (1.1)$$

m: the number of principal components (PCs);
x_j: the jth PC;
q_j: the intercept of the regression of jth PC on biological age (BA), since BA is unknown, it is replaced by chronological age (CA), the same as below;
k_j: the slope of the regression of jth PC on BA;
s_j: the root mean square error of the regression of jth PC on BA;
s_{BA}: the root mean square error of the regression of CA on BA, and it can be replaced by the root mean square error of the regression of all PCs on CA account of unknown BA;

$$\mathrm{KDM-BA} = \beta_0 + \beta_1 * \mathrm{CA} + u \quad (1.2)$$

The regression residual (u) represents Klemera-Doubal's Biological Age Acceleration (KDM-BAacc)

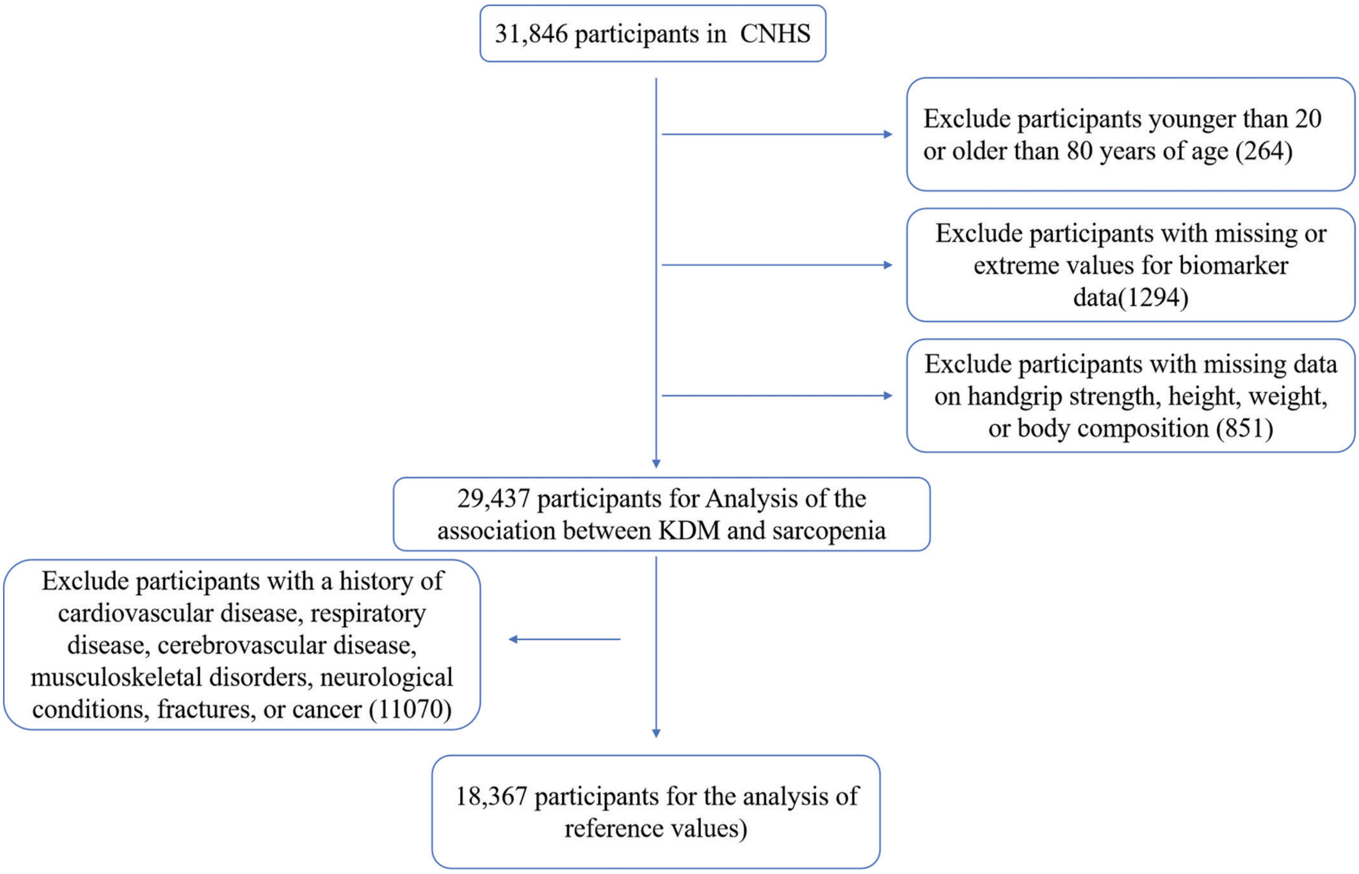

FIG. S1. Flowchart for the selection of study participants
To ensure that participants were old enough to be experiencing detectable age-related changes in biomarkers, but not too old as to represent a selected group with above-(or below-) average health (eg, the oldest old ≥ 80 years), we restricted study participants to those aged between 20 and 79 years.

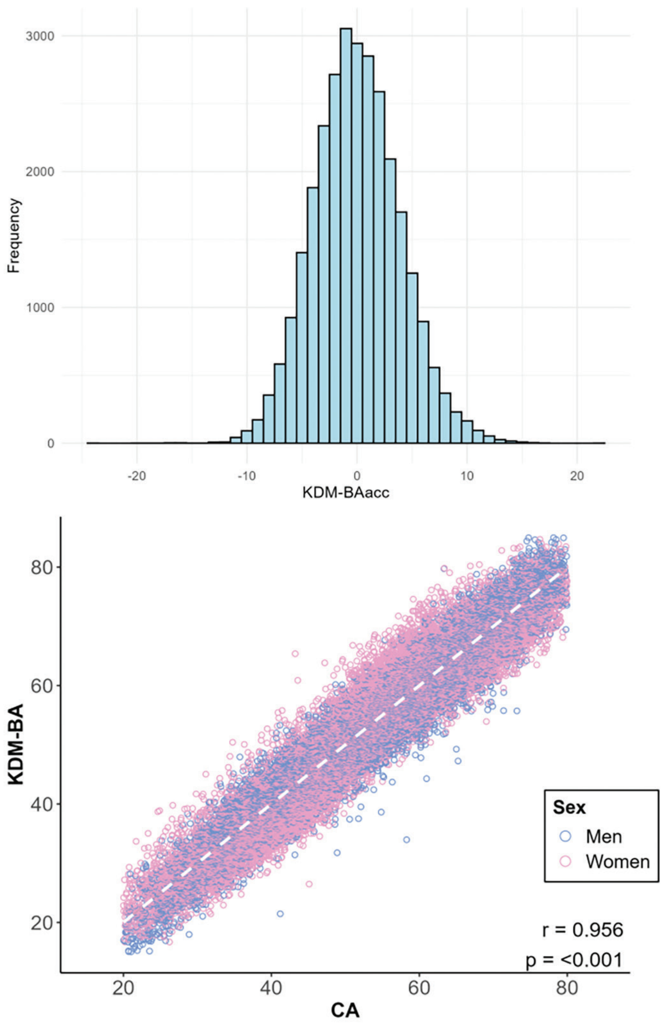

FIG. S2. Characteristics of KDM-BA, KDM-BAacc
KDM-BA = Klemera and Doubal method-biological age; KDM-BAacc = Klemera and Doubal method-biological age acceleration; CA = chronological age. The figure shows the distribution of KDM-BAacc and the correlation between CA and the KDM-BA.

TABLE S1. Summary of Reagents, Calibrators, and Instrument Settings for Clinical Biochemistry Assays

Biomarkers	Reagent	Reaction type	Analytical wavelength	Calibrator
Albumin, g/L	Beckman reagent: REF OSR6102	End point (Bromocresol Green)	Primary: 600 nm Secondary: 800 nm	Beckman Coulter System Calibrator: REF 66300
Alkaline phosphatase,U/L	Beckman reagent: REF OSR6104	Rate (IFCC)	Primary: 410 nm Secondary: 480 nm	Beckman Coulter System Calibrator: REF 66300
Alanine aminotransferase,U/L	Beckman reagent: REF OSR6107	Rate (IFCC)	Primary: 340 nm Secondary: 660 nm	Beckman Coulter System Calibrator: REF 66300
Aspartate aminotransferase, U/L	Beckman reagent: REF OSR6109	Rate (IFCC)	Primary: 340 nm Secondary: 660 nm	Beckman Coulter System Calibrator: REF 66300
Total cholesterol, mmol/L	Beckman reagent: REF OSR6116	End point (Cholesterol Oxidase)	Primary: 540 nm Secondary: 600 nm	Beckman Coulter System Calibrator: REF 66300
Creatinine, umol/L	Beckman reagent: REF OSR61204	End point (Enzymatic)	Primary: 600 nm Secondary: 700 nm	Beckman Coulter System Calibrator: REF 66300
Fasting glucose, mmol/L	Beckman reagent: REF OSR6221	End point (hexokinase method)	Primary: 340 nm Secondary: 660 nm	Beckman Coulter System Calibrator: REF 66300
Gamma-glutamyl transferase,U/L	Beckman reagent: REF OSR6120	Rate (IFCC)	Primary: 410 nm Secondary: 480 nm	Beckman Coulter System Calibrator: REF 66300
High density lipoprotein cholesterol, mmol/L	Mindray HDL-Cholesterol Assay Kit (Selective Inhibition Method), Lot: 591AEA	End point	Primary: 600 nm Secondary: 700 nm	Mindray HDL/LDL Cholesterol Calibrator, Lot: 612ADL
Low density lipoprotein cholesterol, mmol/L	Mindray LDL-Cholesterol Assay Kit (Soluble Reaction Method), Lot: 577ADL	End point	Primary: 600 nm Secondary: 700 nm	Mindray HDL/LDL Cholesterol Calibrator, Lot: 612ADL
Lymphocyte absolute Count, × 109/L	Lysercell WDF; Fluorocell WDF	Flow cytometry using semiconductor laser	633 nm	Not applicable
Platelet count, × 109/L	CELLPACK DCL	Hydraulic focusing method (DC detection)	Not applicable	XN CAL
Red blood cell count, × 1012/L	CELLPACK DCL	Hydraulic focusing method (DC detection)	Not applicable	XN CAL
Total triglyceride, mmol/L	Beckman reagent: REF OSR61118	End point (GPO-POD)	Primary: 660 nm Secondary: 800 nm	Beckman Coulter System Calibrator: REF 66300
Uric acid, umol/L	Beckman reagent: REF OSR6298	End point (Uricase peroxidase)	Primary: 660 nm Secondary: 800 nm	Beckman Coulter System Calibrator: REF 66300
Urea,mmol/L	Beckman reagent: REF OSR6134	Rate (Urease kinetic)	Primary: 340 nm Secondary: 660 nm	Beckman Coulter System Calibrator: REF 66300
White blood cell count, × 109/L	Lysercell WNR; Fluorocell WNR	Flow cytometry using semiconductor laser	633 nm	XN CAL

The asterisk (*) in reagent reference codes indicates values that vary by reagent pack size

TABLE S2. Sex-stratified Pearson correlations between chronological age and biomarkers or anthropometric measures

	Male		Female	
	**r**	***P*-value**	**r**	***P*-value**
	Biomarkers			
Albumin, g/L	**-0.33**	2.9738E-268	-0.06	2.37434E-14
Alkaline phosphatase,U/L	0.04	5.91678E-06	**0.47**	0.000000e+00
Alanine aminotransferase,U/L	**-0.29**	1.1858E-204	**0.17**	1.5864E-118
Aspartate aminotransferase, U/L	-0.03	0.000719207	**0.31**	0.000000e+00
Total cholesterol, mmol/L	-0.07	9.05074E-12	**0.25**	9.1092E-262
Creatinine, umol/L	0.03	0.000389082	**0.16**	1.5392E-107
Fasting glucose, mmol/L	**0.22**	1.5952E-121	**0.33**	0.000000e+00
Gamma-glutamyl transferase,U/L	**-0.15**	3.12788E-56	**0.18**	1.6725E-142
High density lipoprotein cholesterol, mmol/L	**0.16**	9.55834E-62	-0.03	1.02869E-05
Low density lipoprotein cholesterol, mmol/L	-0.05	2.11701E-08	**0.21**	2.3111E-184
Lymphocyte absolute Count, $\times 10^9$/L	**-0.22**	7.9871E-118	0.01	0.343109569
Platelet count, $\times 10^9$/L	**-0.26**	7.4273E-163	**-0.20**	1.6135E-165
Red blood cell count, $\times 10^{12}$/L	**-0.35**	2.1059E-318	-0.08	1.70119E-27
Total triglyceride, mmol/L	**-0.14**	8.21611E-51	**0.27**	3.783E-308
Uric acid, umol/L	**-0.23**	1.3446E-132	0.06	7.97783E-15
Urea,mmol/L	**0.29**	5.6452E-203	**0.40**	0.000000e+00
White blood cell count, $\times 10^9$/L	-0.09	9.58808E-23	-0.04	8.82271E-09
Systolic blood pressure, mmHg	**0.26**	4.5389E-163	**0.48**	0.000000e+00
Diastolic blood pressure, mmHg	-0.06	1.03066E-10	**0.15**	2.3373E-89

r = Pearson correlations

TABLE S3. Distribution of appendicular skeletal muscle mass and appendicular skeletal muscle mass index, stratified by sex and bioage

	Male								Female							
	n	Centiles					Mean	SD	n	Centiles					Mean	SD
		5th	25th	50th	75th	95th				5th	25th	50th	75th	95th		
**ASM (kg)**																
**Age groups**																
**< 20**	**61**	21.50	24.50	26.3	28.70	33.00	26.59	3.54	**43**	15.40	16.9	17.90	18.65	20.60	17.93	1.71
**20-**	**159**	20.84	24.10	26.2	28.85	34.60	26.64	3.86	**241**	14.70	16.3	17.30	18.20	19.90	17.29	1.52
**25-**	**260**	20.50	23.98	26.6	29.25	34.71	27.06	4.34	**609**	14.80	16.0	17.30	18.40	20.62	17.42	1.88
**30-**	**403**	20.63	24.20	26.6	28.75	33.49	26.79	3.80	**1 030**	14.60	16.0	17.20	18.40	20.40	17.33	1.83
**35-**	**485**	20.90	23.90	26.2	28.90	33.26	26.46	3.81	**1 201**	14.70	16.1	17.30	18.50	20.90	17.46	1.91
**40-**	**674**	20.30	23.30	25.6	28.30	32.50	25.95	3.72	**1 183**	14.50	15.9	17.20	18.50	20.79	17.34	1.97
**45-**	**783**	19.80	22.60	24.8	27.70	31.89	25.20	3.66	**1 114**	14.50	15.7	16.95	18.20	20.43	17.09	1.91
**50-**	**897**	19.10	21.90	24.3	26.50	30.32	24.39	3.48	**1 358**	13.90	15.2	16.50	17.90	20.30	16.71	2.04
**55-**	**960**	18.90	21.50	24.0	26.20	29.90	24.04	3.44	**1 694**	13.50	15.1	16.20	17.50	19.40	16.35	1.84
**60-**	**860**	18.50	21.28	23.2	25.50	29.20	23.45	3.24	**1 481**	13.00	14.8	15.90	17.10	19.10	15.99	1.87
**65-**	**603**	18.02	20.60	22.6	24.70	27.69	22.72	2.99	**1 028**	12.60	14.4	15.50	16.80	18.60	15.61	1.86
**70-**	**378**	17.37	19.80	21.9	23.78	27.23	21.96	3.16	**499**	12.30	14.2	15.30	16.60	18.71	15.36	1.91
**75-**	**170**	15.85	19.62	21.6	23.87	26.65	21.63	3.13	**133**	11.92	13.4	14.60	15.80	17.68	14.66	1.78
**> 80**	**39**	17.36	19.30	21.4	23.80	25.25	21.54	2.85	**21**	12.80	13.8	14.50	15.50	16.10	14.56	1.36
**Overall**	**6 732**	18.90	21.90	24.3	26.90	31.20	24.57	3.83	**11 635**	13.60	15.3	16.60	17.90	20.10	16.67	2.02
ASMI (ASM/BMI, m^2)																
Age groups																
**< 20**	**61**	1.03	1.11	1.18	1.25	1.40	1.19	0.12	**43**	0.72	0.82	0.85	0.91	1.00	0.86	0.09
**20-**	**159**	0.94	1.03	1.10	1.17	1.31	1.10	0.11	**241**	0.71	0.78	0.82	0.89	0.98	0.83	0.08
**25-**	**260**	0.93	1.01	1.07	1.14	1.24	1.08	0.10	**609**	0.67	0.74	0.80	0.86	0.93	0.80	0.08
**30-**	**403**	0.90	0.98	1.05	1.12	1.23	1.05	0.10	**1 030**	0.64	0.71	0.76	0.82	0.89	0.76	0.08
**35-**	**485**	0.88	0.97	1.03	1.10	1.19	1.03	0.09	**1 201**	0.63	0.69	0.74	0.79	0.86	0.74	0.07
**40-**	**674**	0.86	0.94	1.00	1.06	1.15	1.00	0.09	**1 183**	0.59	0.66	0.71	0.76	0.84	0.72	0.08
**45-**	**783**	0.85	0.92	0.98	1.05	1.15	0.99	0.10	**1 114**	0.59	0.65	0.70	0.74	0.81	0.70	0.07
**50-**	**897**	0.83	0.90	0.97	1.03	1.13	0.97	0.09	**1 358**	0.57	0.63	0.68	0.73	0.80	0.68	0.07
**55-**	**960**	0.81	0.89	0.95	1.01	1.11	0.95	0.09	**1 694**	0.56	0.62	0.66	0.71	0.78	0.67	0.07
**60-**	**860**	0.81	0.89	0.95	1.00	1.09	0.95	0.09	**1 481**	0.54	0.61	0.65	0.69	0.76	0.65	0.07
**65-**	**603**	0.79	0.86	0.91	0.97	1.06	0.92	0.08	**1 028**	0.53	0.58	0.63	0.67	0.75	0.63	0.07
**70-**	**378**	0.78	0.85	0.90	0.95	1.04	0.90	0.08	**499**	0.50	0.56	0.60	0.66	0.72	0.61	0.07
**75-**	**170**	0.74	0.83	0.88	0.93	1.03	0.88	0.08	**133**	0.49	0.54	0.58	0.62	0.70	0.58	0.06
**> 80**	**39**	0.76	0.80	0.85	0.90	0.96	0.86	0.08	**21**	0.51	0.55	0.58	0.59	0.63	0.57	0.04
**Overall**	**6 732**	0.81	0.90	0.97	1.04	1.16	0.98	0.11	**11 635**	0.56	0.63	0.69	0.75	0.85	0.70	0.09

TABLE S4. Distribution of absolute and relative hand grip strength, stratified by sex and bioage

	Male								Female							
	n	Centiles					Mean	SD	n	Centiles					Mean	SD
		5th	25th	50th	75th	95th				5th	25th	50th	75th	95th		
**Absolute Grip Strength (kg)**																
**Age groups**																
**< 20**	**61**	35.70	40.70	49.80	54.40	58.00	48.07	8.27	**43**	19.70	23.85	27.30	32.20	38.99	27.70	5.72
**20-**	**159**	34.06	42.50	47.60	52.20	60.33	47.38	7.86	**241**	18.90	24.60	27.80	31.10	35.00	27.85	5.14
**25-**	**260**	35.48	42.60	48.05	54.60	63.12	48.78	8.58	**609**	20.78	25.10	28.20	31.50	37.40	28.58	5.25
**30-**	**403**	34.41	44.05	49.30	54.05	61.60	48.81	8.46	**1 030**	21.20	25.70	28.90	32.40	37.90	29.19	5.12
**35-**	**485**	35.50	43.30	48.00	53.50	60.28	48.14	7.86	**1 201**	20.70	25.60	29.00	32.80	38.40	29.24	5.33
**40-**	**674**	34.17	41.52	47.30	52.80	59.84	47.18	8.10	**1 183**	20.31	25.50	28.80	32.55	38.08	28.98	5.44
**45-**	**783**	31.91	39.90	45.50	51.20	58.19	45.49	8.20	**1 114**	19.10	24.50	28.35	31.60	37.03	28.18	5.30
**50-**	**897**	30.88	38.70	43.80	49.20	56.80	43.91	7.90	**1 358**	19.00	23.50	26.80	30.20	35.60	26.96	5.05
**55-**	**960**	28.60	37.00	42.55	47.70	55.50	42.39	8.08	**1 694**	18.60	23.10	26.40	29.60	35.30	26.47	5.06
**60-**	**860**	29.20	36.10	41.40	46.40	54.00	41.43	7.77	**1 481**	17.90	22.50	25.50	29.00	33.60	25.72	4.81
**65-**	**603**	26.82	33.50	38.90	43.65	50.39	38.65	7.44	**1 028**	16.54	21.40	24.40	28.10	32.20	24.60	4.88
**70-**	**378**	23.80	31.90	36.60	41.77	49.22	36.78	7.67	**499**	15.48	20.00	23.30	26.60	31.20	23.35	4.98
**75-**	**170**	24.02	30.52	35.25	39.70	46.30	34.87	6.97	**133**	15.80	18.80	21.30	24.10	28.40	21.57	4.06
**> 80**	**39**	24.41	30.50	35.20	39.90	44.34	34.91	6.47	**21**	18.50	20.40	21.40	25.80	28.10	22.86	3.31
**Overall**	**6 732**	29.50	37.60	43.60	49.50	58.10	43.64	8.80	**11 635**	18.60	23.50	27.00	30.60	36.30	27.16	5.42
**Relative Grip Strength (Absolute Grip Strength/BMI,m^2)**																
Age groups																
**< 20**	**61**	1.52	1.86	2.16	2.45	3.03	2.18	0.46	**43**	0.86	1.16	1.36	1.50	1.70	1.33	0.28
**20-**	**159**	1.30	1.70	2.02	2.25	2.71	1.99	0.44	**241**	0.88	1.19	1.35	1.52	1.75	1.35	0.27
**25-**	**260**	1.26	1.72	1.98	2.25	2.78	1.99	0.45	**609**	0.92	1.13	1.30	1.49	1.75	1.32	0.27
**30-**	**403**	1.23	1.68	1.93	2.21	2.58	1.94	0.42	**1 030**	0.90	1.11	1.29	1.46	1.74	1.30	0.26
**35-**	**485**	1.26	1.66	1.90	2.15	2.61	1.91	0.39	**1 201**	0.84	1.08	1.24	1.42	1.68	1.25	0.26
**40-**	**674**	1.26	1.57	1.83	2.09	2.47	1.84	0.37	**1 183**	0.81	1.02	1.19	1.37	1.65	1.20	0.26
**45-**	**783**	1.23	1.55	1.80	2.06	2.38	1.80	0.37	**1 114**	0.78	0.98	1.16	1.33	1.58	1.16	0.25
**50-**	**897**	1.20	1.51	1.74	2.00	2.40	1.76	0.36	**1 358**	0.74	0.94	1.10	1.26	1.50	1.11	0.24
**55-**	**960**	1.12	1.45	1.67	1.92	2.34	1.69	0.36	**1 694**	0.73	0.92	1.08	1.24	1.48	1.09	0.24
**60-**	**860**	1.14	1.46	1.68	1.91	2.27	1.69	0.35	**1 481**	0.70	0.91	1.04	1.20	1.42	1.05	0.22
**65-**	**603**	1.03	1.35	1.57	1.80	2.15	1.58	0.34	**1 028**	0.65	0.85	0.98	1.14	1.38	1.00	0.23
**70-**	**378**	0.99	1.28	1.52	1.77	2.08	1.52	0.34	**499**	0.58	0.78	0.93	1.08	1.32	0.93	0.23
**75-**	**170**	0.97	1.22	1.43	1.64	1.87	1.43	0.30	**133**	0.62	0.72	0.84	0.95	1.26	0.87	0.20
**> 80**	**39**	0.90	1.29	1.40	1.53	1.82	1.39	0.26	**21**	0.66	0.77	0.88	1.02	1.16	0.90	0.16
**Overall**	**6 732**	1.13	1.48	1.74	2.00	2.43	1.76	0.40	**11 635**	0.73	0.95	1.13	1.31	1.61	1.14	0.27

TABLE S5. Associations between KDM-BAacc and muscle-related indicators (weight-adjusted,fully adjusted model)[a]

Biological age	ASMI		Relative Grip Strength		LMM			LMS			Sarcopenia		
	Coefficients (SE)	p-value	Coefficients (SE)	p-value	N_case/N_total	Odds ratio (95% CI)	p-value	N_case/N_total	Odds ratio (95% CI)	p-value	N_case/N_total	Odds ratio (95% CI)	p-value
KDM-BAacc (Male)[b]	-0.0022 (0.0001)	< 0.0001	-0.0046 (0.0003)	< 0.0001	2152/10735	2.28 (2.06–2.53)	< 0.0001	2152/10735	1.48 (1.35–1.64)	< 0.0001	795/10735	2.21 (1.89–2.58)	< 0.0001
**KDM-BAacc (Quartile)**													
Q1	Ref		Ref		294/2684	Ref		427/2684	Ref		95/2684	Ref	
Q2	-0.0095 (0.0006)	< 0.0001	-0.0221 (0.0033)	< 0.0001	448/2684	1.72 (1.47–2.03)	< 0.0001	507/2684	1.31 (1.13–1.51)	3e-04	166/2684	1.9 (1.47–2.47)	< 0.0001
Q3	-0.0157 (0.0006)	< 0.0001	-0.0296 (0.0033)	< 0.0001	594/2684	2.44 (2.09–2.86)	< 0.0001	561/2684	1.53 (1.33–1.77)	< 0.0001	230/2684	2.69 (2.11–3.46)	< 0.0001
Q4	-0.0210 (0.0006)	< 0.0001	-0.0481 (0.0034)	< 0.0001	816/2683	3.83 (3.3–4.46)	< 0.0001	657/2683	1.9 (1.65–2.19)	< 0.0001	304/2683	3.7 (2.92–4.73)	< 0.0001
KDM-BAacc (Female)	-0.0018 (0.0000)	< 0.0001	-0.0027 (0.0002)	< 0.0001	3746/18702	2.09 (1.93–2.26)	< 0.0001	3747/18702	1.35 (1.25–1.46)	< 0.0001	1563/18702	1.93 (1.72–2.17)	< 0.0001
**KDM-BAacc (Quartile)**													
Q1	Ref		Ref		440/4676	Ref		728/4676	Ref		188/4676	Ref	
Q2	-0.0074 (0.0005)	< 0.0001	-0.0078 (0.0019)	< 0.0001	783/4676	1.87 (1.65–2.13)	< 0.0001	828/4676	1.12 (1–1.25)	0.0432	306/4676	1.59 (1.32–1.92)	< 0.0001
Q3	-0.0112 (0.0005)	< 0.0001	-0.0147 (0.0019)	< 0.0001	972/4675	2.31 (2.04–2.62)	< 0.0001	953/4675	1.28 (1.15–1.43)	< 0.0001	393/4675	1.96 (1.64–2.36)	< 0.0001
Q4	-0.0181 (0.0005)	< 0.0001	-0.0268 (0.0021)	< 0.0001	1551/4675	4.05 (3.58–4.59)	< 0.0001	1238/4675	1.62 (1.45–1.81)	< 0.0001	676/4675	3.21 (2.69–3.85)	< 0.0001

[a]Fully adjusted for age, socioeconomic status (residence, education level, marital status), lifestyle behaviors (smoking, drinking, physical activity), and clinical comorbidities (hypertension and diabetes)

[b]For ASMI and relative grip strength, KDM-BAacc was modeled as a continuous variable (per 1-year increase) in linear regression models. For LMM, LMS, and sarcopenia, KDM-BAacc was dichotomized (accelerated vs. non-accelerated aging) in logistic regression models, with all outcomes treated as binary variables.

TABLE S6. Associations between KDM-BAacc and muscle-related indicators (healthy reference population[a],fully adjusted model[b])

Biological age	ASMI		Relative Grip Strength		LMM			LMS			Sarcopenia		
	Coefficients (SE)	p-value	Coefficients (SE)	p-value	N_case/N_total	Odds ratio (95% CI)	p-value	N_case/N_total	Odds ratio (95% CI)	p-value	N_case/N_total	Odds ratio (95% CI)	p-value
KDM-BAacc (Male)[b]	-0.0064 (0.0003)	< 0.0001	-0.0146 (0.0012)	< 0.0001	1374/6732	1.75 (1.55–1.99)	< 0.0001	1268/6732	1.38 (1.21–1.57)	< 0.0001	552/6732	1.74 (1.45–2.1)	< 0.0001
KDM-BAacc (Quartile)													
Q1	Ref		Ref		250/1721	Ref		271/1721	Ref		94/1721	Ref	
Q2	-0.0275 (0.0031)	< 0.0001	-0.0676 (0.0124)	< 0.0001	310/1688	1.38 (1.15–1.66)	0.0007	309/1688	1.27 (1.06–1.53)	0.0099	126/1688	1.51 (1.14–2)	0.0045
Q3	-0.0414 (0.0032)	< 0.0001	-0.0858 (0.0125)	< 0.0001	351/1658	1.72 (1.43–2.07)	< 0.0001	295/1658	1.29 (1.07–1.56)	0.0072	134/1658	1.72 (1.3–2.28)	0.0002
Q4	-0.0620 (0.0032)	< 0.0001	-0.1601 (0.0126)	< 0.0001	463/1665	2.48 (2.07–2.96)	< 0.0001	393/1665	1.86 (1.56–2.23)	< 0.0001	198/1665	2.66 (2.05–3.48)	< 0.0001
KDM-BAacc (Female)	-0.0046 (0.0002)	< 0.0001	-0.0078 (0.0006)	< 0.0001	2381/11635	1.94 (1.75–2.15)	< 0.0001	2271/11635	1.47 (1.33–1.63)	< 0.0001	1130/11635	2.16 (1.87–2.49)	< 0.0001
KDM-BAacc (Quartile)													
Q1	Ref		Ref		328/2886	Ref		412/2886	Ref		142/2886	Ref	
Q2	-0.0167 (0.0018)	< 0.0001	-0.0146 (0.0063)	0.0209	490/2971	1.46 (1.25–1.7)	< 0.0001	481/2971	1.1 (0.95–1.27)	0.2084	192/2971	1.21 (0.97–1.53)	0.0967
Q3	-0.0277 (0.0019)	< 0.0001	-0.0410 (0.0065)	< 0.0001	607/2857	1.86 (1.59–2.16)	< 0.0001	569/2857	1.35 (1.17–1.56)	< 0.0001	284/2857	1.82 (1.47–2.26)	< 0.0001
Q4	-0.0468 (0.0020)	< 0.0001	-0.0798 (0.0069)	< 0.0001	956/2921	3.02 (2.59–3.52)	< 0.0001	809/2921	1.86 (1.61–2.15)	< 0.0001	512/2921	3.08 (2.5–3.81)	< 0.0001

[a]The “healthy reference population” refers to a relatively healthy subsample used for constructing LMS reference curves for muscle indicators.

[b]Fully adjusted for age, socioeconomic status (residence, education level, marital status), lifestyle behaviors (smoking, drinking, physical activity), and clinical comorbidities (hypertension and diabetes)

[c]For ASMI and relative grip strength, KDM-BAacc was modeled as a continuous variable (per 1-year increase) in linear regression models. For LMM, LMS, and sarcopenia, KDM-BAacc was dichotomized (accelerated vs. non-accelerated aging) in logistic regression models, with all outcomes treated as binary variables.

TABLE S7. Associations between KDM-BAacc and muscle-related indicators (non-healthy reference population[a],fully adjusted model[b])

Biological age	ASMI		Relative Grip Strength		LMM			LMS			Sarcopenia		
	Coefficients (SE)	p-value	Coefficients (SE)	p-value	N_case/N_total	Odds ratio (95% CI)	p-value	N_case/N_total	Odds ratio (95% CI)	p-value	N_case/N_ total	Odds ratio (95% CI)	p-value
KDM-BAacc (Male)[c]	-0.0062 (0.0004)	< 0.0001	-0.0106 (0.0016)	< 0.0001	778/4003	1.68 (1.43–1.98)	< 0.0001	884/4003	1.27 (1.09–1.48)	0.0026	340/4003	1.55 (1.23–1.96)	0.0002
KDM-BAacc (Quartile)													
Q1	Ref		Ref		133/963	Ref		173/963	Ref		48/963	Ref	
Q2	-0.0291 (0.0042)	< 0.0001	-0.0589 (0.0161)	0.0003	174/996	1.45 (1.13–1.87)	0.0039	228/996	1.48 (1.18–1.85)	0.0008	86/996	1.91 (1.33–2.78)	0.0006
Q3	-0.0460 (0.0042)	< 0.0001	-0.0757 (0.0160)	< 0.0001	221/1026	1.92 (1.51–2.45)	< 0.0001	238/1026	1.53 (1.22–1.92)	0.0002	93/1026	2.02 (1.41–2.94)	0.0002
Q4	-0.0586 (0.0042)	< 0.0001	-0.1059 (0.0162)	< 0.0001	250/1018	2.23 (1.76–2.84)	< 0.0001	245/1018	1.6 (1.28–2.01)	< 0.0001	113/1018	2.5 (1.76–3.6)	< 0.0001
KDM-BAacc (Female)	-0.0035 (0.0002)	< 0.0001	-0.0047 (0.0007)	< 0.0001	1365/7067	1.54 (1.35–1.76)	< 0.0001	1475/7067	1.24 (1.09–1.41)	0.0008	659/7067	1.32 (1.1–1.57)	0.0023
KDM-BAacc (Quartile)													
Q1	Ref		Ref		195/1790	Ref		295/1790	Ref		103/1790	Ref	
Q2	-0.0128 (0.0024)	< 0.0001	-0.0169 (0.0079)	0.0321	312/1705	1.71 (1.4–2.08)	< 0.0001	334/1705	1.14 (0.95–1.36)	0.1585	146/1705	1.36 (1.04–1.78)	0.0237
Q3	-0.0215 (0.0024)	< 0.0001	-0.0238 (0.0079)	0.0027	370/1818	1.83 (1.5–2.22)	< 0.0001	400/1818	1.25 (1.05–1.49)	0.0121	175/1818	1.42 (1.1–1.85)	0.0086
Q4	-0.0344 (0.0025)	< 0.0001	-0.0420 (0.0084)	< 0.0001	488/1754	2.56 (2.11–3.13)	< 0.0001	446/1754	1.32 (1.1–1.59)	0.0026	235/1754	1.79 (1.38–2.33)	< 0.0001

[a]The “non-reference population” refers to participants who were not included in the LMS-based healthy reference sample, often due to the presence of chronic conditions, functional impairments, or other exclusion criteria.

[b]Fully adjusted for age, socioeconomic status (residence, education level, marital status), lifestyle behaviors (smoking, drinking, physical activity), and clinical comorbidities (hypertension and diabetes)

[c]For ASMI and relative grip strength, KDM-BAacc was modeled as a continuous variable (per 1-year increase) in linear regression models. For LMM, LMS, and sarcopenia, KDM-BAacc was dichotomized (accelerated vs. non-accelerated aging) in logistic regression models, with all outcomes treated as binary variables.

TABLE S8. Associations between KDM-BAacc and muscle-related indicators (total population,fully adjusted model)[a]

Biological age	ASMI		Relative Grip Strength		LMM			LMS			Sarcopenia		
	Coefficients (SE)	p-value	Coefficients (SE)	p-value	N_case/N_total	Odds ratio (95% CI)	p-value	N_case/N_total	Odds ratio (95% CI)	p-value	N_case/N_total	Odds ratio (95% CI)	p-value
KDM-BAac[b]	-0.0047 (0.0001)	< 0.0001	-0.0073 (0.0004)	< 0.0001	5898/29437	1.76 (1.66–1.87)	< 0.0001	5898/29437	1.32 (1.25–1.4)	< 0.0001	2681/29437	1.73 (1.59–1.88)	< 0.0001
**KDM-BAacc (Quartile)**													
Q1	Ref		Ref		906/7360	Ref		1151/7360	Ref		387/7360	Ref	
Q2	-0.0195 (0.0013)	< 0.0001	-0.0299 (0.0048)	< 0.0001	1286/7360	1.48 (1.35–1.63)	< 0.0001	1352/7360	1.19 (1.09–1.29)	0.0002	550/7360	1.39 (1.22–1.6)	< 0.0001
Q3	-0.0311 (0.0013)	< 0.0001	-0.0452 (0.0048)	< 0.0001	1549/7359	1.81 (1.65–1.98)	< 0.0001	1502/7359	1.31 (1.2–1.43)	< 0.0001	686/7359	1.69 (1.49–1.93)	< 0.0001
Q4	-0.0473 (0.0013)	< 0.0001	-0.0795 (0.0049)	< 0.0001	2157/7358	2.61 (2.39–2.86)	< 0.0001	1893/7358	1.59 (1.46–1.73)	< 0.0001	1058/7358	2.48 (2.19–2.82)	< 0.0001

[a]Fully adjusted for age, socioeconomic status (residence, education level, marital status), lifestyle behaviors (smoking, drinking, physical activity), and clinical comorbidities (hypertension and diabetes)

[b]For ASMI and relative grip strength, KDM-BAacc was modeled as a continuous variable (per 1-year increase) in linear regression models. For LMM, LMS, and sarcopenia, KDM-BAacc was dichotomized (accelerated vs. non-accelerated aging) in logistic regression models, with all outcomes treated as binary variables.

TABLE S9. Associations between KDM-BAacc and muscle-related indicators (age ≥ 60, fully adjusted model)[a]

Biological age	ASMI		Relative Grip Strength		LMM			LMS			Sarcopenia		
	Coefficients (SE)	p-value	Coefficients (SE)	p-value	N_case/N_total	Odds ratio (95% CI)	p-value	N_case/N_total	Odds ratio (95% CI)	p-value	N_case/N_total	Odds ratio (95% CI)	p-value
KDM-BAacc (Male)[b]	-0.0055 (0.0004)	< 0.0001	-0.0088 (0.0014)	< 0.0001	843/4209	1.62 (1.38–1.9)	< 0.0001	844/4209	1.16 (0.99–1.35)	0.0689	320/4209	1.43 (1.13–1.82)	0.0029
**KDM-BAacc (Quartile)**													
Q1	Ref		Ref		167/1077	Ref		203/1077	Ref		59/1077	Ref	
Q2	-0.0222 (0.0039)	< 0.0001	-0.0482 (0.0146)	0.001	186/1043	1.29 (1.02–1.63)	0.0333	218/1043	1.27 (1.02–1.58)	0.0361	84/1043	1.72 (1.21–2.45)	0.0026
Q3	-0.0379 (0.0039)	< 0.0001	-0.0614 (0.0146)	< 0.0001	224/1075	1.63 (1.3–2.04)	< 0.0001	205/1075	1.22 (0.97–1.53)	0.0825	78/1075	1.64 (1.15–2.35)	0.0071
Q4	-0.0527 (0.0040)	< 0.0001	-0.0969 (0.0148)	< 0.0001	266/1014	2.13 (1.71–2.67)	< 0.0001	218/1014	1.4 (1.12–1.76)	0.0029	99/1014	2.25 (1.6–3.2)	< 0.0001
KDM-BAacc (Female)	-0.0028 (0.0002)	< 0.0001	-0.0031 (0.0008)	0.0001	1274/6365	1.41 (1.23–1.62)	< 0.0001	1275/6365	1.17 (1.02–1.34)	0.0264	577/6365	1.35 (1.11–1.63)	0.0022
**KDM-BAacc (Quartile)**													
Q1	Ref		Ref		253/1758	Ref		313/1758	Ref		124/1758	Ref	
Q2	-0.0063 (0.0023)	0.0059	0.0042 (0.0077)	0.5898	314/1645	1.34 (1.11–1.62)	0.0028	302/1645	0.95 (0.79–1.14)	0.5571	132/1645	1.04 (0.8–1.35)	0.7796
Q3	-0.0139 (0.0024)	< 0.0001	-0.0077 (0.0080)	0.3371	324/1597	1.39 (1.15–1.69)	0.0008	321/1597	1.03 (0.85–1.24)	0.7622	147/1597	1.18 (0.91–1.54)	0.2153
Q4	-0.0261 (0.0026)	< 0.0001	-0.0245 (0.0089)	0.006	383/1365	2.08 (1.7–2.55)	< 0.0001	339/1365	1.21 (0.99–1.48)	0.0563	174/1365	1.58 (1.2–2.08)	0.0011

[a]Fully adjusted for age, socioeconomic status (residence, education level, marital status), lifestyle behaviors (smoking, drinking, physical activity), and clinical comorbidities (hypertension and diabetes)

[b]For ASMI and relative grip strength, KDM-BAacc was modeled as a continuous variable (per 1-year increase) in linear regression models. For LMM, LMS, and sarcopenia, KDM-BAacc was dichotomized (accelerated vs. non-accelerated aging) in logistic regression models, with all outcomes treated as binary variables.

TABLE S10. Associations between KDM-BAacc and muscle-related indicators (age < 60, fully adjusted model)[a]

Biological age	ASMI		Relative Grip Strength		LMM			LMS			Sarcopenia		
	Coefficients (SE)	p-value	Coefficients (SE)	p-value	N_case/N_total	Odds ratio (95% CI)	p-value	N_case/N_total	Odds ratio (95% CI)	p-value	N_case/N_total	Odds ratio (95% CI)	p-value
KDM-BAacc (Male)[b]	-0.0069 (0.0003)	< 0.0001	-0.0166 (0.0013)	< 0.0001	1309/6526	1.81 (1.59–2.06)	< 0.0001	1308/6526	1.47 (1.3–1.67)	< 0.0001	572/6526	1.85 (1.55–2.23)	< 0.0001
KDM-BAacc (Quartile)													
Q1	Ref		Ref		216/1607	Ref		241/1607	Ref		83/1607	Ref	
Q2	-0.0314 (0.0033)	< 0.0001	-0.0789 (0.0131)	< 0.0001	298/1641	1.49 (1.22–1.81)	0.0001	319/1641	1.42 (1.18–1.72)	0.0002	128/1641	1.61 (1.2–2.16)	0.0014
Q3	-0.0463 (0.0033)	< 0.0001	-0.1023 (0.0132)	< 0.0001	348/1609	1.92 (1.59–2.33)	< 0.0001	328/1609	1.54 (1.27–1.86)	< 0.0001	149/1609	1.98 (1.5–2.65)	< 0.0001
Q4	-0.0654 (0.0033)	< 0.0001	-0.1721 (0.0131)	< 0.0001	447/1669	2.58 (2.15–3.11)	< 0.0001	420/1669	2.03 (1.7–2.44)	< 0.0001	212/1669	2.86 (2.19–3.77)	< 0.0001
KDM-BAacc (Female)	-0.0050 (0.0002)	< 0.0001	-0.0091 (0.0006)	< 0.0001	2472/12337	2.14 (1.93–2.37)	< 0.0001	2471/12337	1.55 (1.41–1.71)	< 0.0001	1212/12337	2.21 (1.92–2.55)	< 0.0001
KDM-BAacc (Quartile)													
Q1	Ref		Ref		270/2918	Ref		394/2918	Ref		121/2918	Ref	
Q2	-0.0207 (0.0018)	< 0.0001	-0.0288 (0.0064)	< 0.0001	488/3031	1.81 (1.54–2.13)	< 0.0001	513/3031	1.26 (1.09–1.46)	0.0017	206/3031	1.57 (1.25–1.99)	0.0002
Q3	-0.0322 (0.0019)	< 0.0001	-0.0541 (0.0065)	< 0.0001	653/3078	2.39 (2.05–2.81)	< 0.0001	648/3078	1.58 (1.37–1.83)	< 0.0001	312/3078	2.27 (1.82–2.84)	< 0.0001
Q4	-0.0510 (0.0020)	< 0.0001	-0.0934 (0.0068)	< 0.0001	1061/3310	3.87 (3.3–4.54)	< 0.0001	916/3310	2.03 (1.76–2.35)	< 0.0001	573/3310	3.75 (3.02–4.69)	< 0.0001

[a]Fully adjusted for age, socioeconomic status (residence, education level, marital status), lifestyle behaviors (smoking, drinking, physical activity), and clinical comorbidities (hypertension and diabetes)

[b]For ASMI and relative grip strength, KDM-BAacc was modeled as a continuous variable (per 1-year increase) in linear regression models. For LMM, LMS, and sarcopenia, KDM-BAacc was dichotomized (accelerated vs. non-accelerated aging) in logistic regression models, with all outcomes treated as binary variables.

TABLE S11. Associations between KDM-BAacc and muscle-related indicators[a]
Unadjusted Model

Biological age	ASMI		Relative Grip Strength		LMM			LMS			Sarcopenia		
	Coefficients (SE)	p-value	Coefficients (SE)	p-value	N_case/N_total	Odds ratio (95% CI)	p-value	N_case/N_total	Odds ratio (95% CI)	p-value	N_case/N_total	Odds ratio (95% CI)	p-value
KDM-BAacc (Male)[a]	-0.0060 (0.0003)	< 0.0001	-0.0113 (0.0010)	< 0.0001	2152/10735	1.63 (1.48–1.79)	< 0.0001	2152/10735	1.24 (1.13–1.37)	< 0.0001	892/10735	1.57 (1.36–1.8)	< 0.0001
**KDM-BAacc (Quartile)**													
Q1	Ref		Ref		383/2684	Ref		444/2684	Ref		142/2684	Ref	
Q2	-0.0269 (0.0029)	< 0.0001	-0.0568 (0.0107)	< 0.0001	484/2684	1.32 (1.14–1.53)	0.0002	537/2684	1.26 (1.1–1.45)	0.001	212/2684	1.54 (1.23–1.92)	0.0001
Q3	-0.0421 (0.0029)	< 0.0001	-0.0720 (0.0107)	< 0.0001	572/2684	1.63 (1.41–1.88)	< 0.0001	533/2684	1.25 (1.09–1.44)	0.0017	227/2684	1.65 (1.33–2.06)	< 0.0001
Q4	-0.0576 (0.0029)	< 0.0001	-0.1236 (0.0107)	< 0.0001	713/2683	2.17 (1.9–2.5)	< 0.0001	638/2683	1.57 (1.38–1.8)	< 0.0001	311/2683	2.35 (1.91–2.89)	< 0.0001
KDM-BAacc (Female)	-0.0050 (0.0002)	< 0.0001	-0.0096 (0.0005)	< 0.0001	3746/18702	2.13 (1.97–2.29)	< 0.0001	3746/18702	1.62 (1.5–1.74)	< 0.0001	1789/18702	2.23 (2.02–2.48)	< 0.0001
**KDM-BAacc (Quartile)**													
Q1	Ref		Ref		523/4676	Ref		707/4676	Ref		245/4676	Ref	
Q2	-0.0169 (0.0018)	< 0.0001	-0.0202 (0.0055)	0.0002	802/4676	1.64 (1.46–1.85)	< 0.0001	815/4676	1.19 (1.06–1.32)	0.0025	338/4676	1.41 (1.19–1.67)	0.0001
Q3	-0.0323 (0.0018)	< 0.0001	-0.0528 (0.0055)	< 0.0001	977/4675	2.1 (1.87–2.35)	< 0.0001	969/4675	1.47 (1.32–1.63)	< 0.0001	459/4675	1.97 (1.68–2.32)	< 0.0001
Q4	-0.0530 (0.0018)	< 0.0001	-0.0999 (0.0055)	< 0.0001	1444/4675	3.55 (3.18–3.96)	< 0.0001	1255/4675	2.06 (1.86–2.28)	< 0.0001	747/4675	3.44 (2.96–4)	< 0.0001

[a]For ASMI and relative grip strength, KDM-BAacc was modeled as a continuous variable (per 1-year increase) in linear regression models. For LMM, LMS, and sarcopenia, KDM-BAacc was dichotomized (accelerated vs. non-accelerated aging) in logistic regression models, with all outcomes treated as binary variables.

Model 1[a]

Biological age	ASMI		Relative Grip Strength		LMM			LMS			Sarcopenia		
	Coefficients (SE)	p-value	Coefficients (SE)	p-value	N_case/N_total	Odds ratio (95% CI)	p-value	N_case/N_total	Odds ratio (95% CI)	p-value	N_case/N_total	Odds ratio (95% CI)	p-value
KDM-BAacc (Male)[b]	-0.0060 (0.0002)	< 0.0001	-0.0113 (0.0010)	< 0.0001	2152/10735	1.62 (1.48–1.79)	< 0.0001	2152/10735	1.24 (1.13–1.37)	< 0.0001	892/10735	1.57 (1.37–1.81)	< 0.0001
**KDM-BAacc (Quartile)**													
Q1	Ref		Ref		383/2684	Ref		444/2684	Ref		142/2684	Ref	
Q2	-0.0255 (0.0025)	< 0.0001	-0.0529 (0.0100)	< 0.0001	484/2684	1.32 (1.14–1.53)	2e-04	537/2684	1.26 (1.1–1.45)	0.0011	212/2684	1.54 (1.24–1.92)	0.0001
Q3	-0.0397 (0.0025)	< 0.0001	-0.0649 (0.0100)	< 0.0001	572/2684	1.63 (1.41–1.88)	< 0.0001	533/2684	1.25 (1.09–1.44)	0.0017	227/2684	1.66 (1.34–2.07)	< 0.0001
Q4	-0.0572 (0.0025)	< 0.0001	-0.1225 (0.0100)	< 0.0001	713/2683	2.17 (1.9–2.5)	< 0.0001	638/2683	1.57 (1.38–1.8)	< 0.0001	311/2683	2.35 (1.92–2.9)	< 0.0001
KDM-BAacc (Female)	-0.0050 (0.0001)	< 0.0001	-0.0096 (0.0004)	< 0.0001	3746/18702	2.12 (1.97–2.29)	< 0.0001	3746/18702	1.62 (1.5–1.74)	< 0.0001	1789/18702	2.24 (2.02–2.48)	< 0.0001
**KDM-BAacc (Quartile)**													
Q1	Ref		Ref		523/4676	Ref		707/4676	Ref		245/4676	Ref	
Q2	-0.0181 (0.0015)	< 0.0001	-0.0227 (0.0050)	< 0.0001	802/4676	1.64 (1.46–1.85)	< 0.0001	815/4676	1.19 (1.06–1.32)	0.0025	338/4676	1.41 (1.19–1.67)	1e-04
Q3	-0.0310 (0.0015)	< 0.0001	-0.0498 (0.0050)	< 0.0001	977/4675	2.1 (1.87–2.35)	< 0.0001	969/4675	1.47 (1.32–1.63)	< 0.0001	459/4675	1.97 (1.68–2.32)	< 0.0001
Q4	-0.0521 (0.0015)	< 0.0001	-0.0979 (0.0050)	< 0.0001	1444/4675	3.55 (3.18–3.96)	< 0.0001	1255/4675	2.06 (1.86–2.28)	< 0.0001	747/4675	3.44 (2.97–4.01)	< 0.0001

[a]Adjusted for age

[b]For ASMI and relative grip strength, KDM-BAacc was modeled as a continuous variable (per 1-year increase) in linear regression models. For LMM, LMS, and sarcopenia, KDM-BAacc was dichotomized (accelerated vs. non-accelerated aging) in logistic regression models, with all outcomes treated as binary variables.

Model 2[a]

Biological age	ASMI		Relative Grip Strength		LMM			LMS			Sarcopenia		
	Coefficients (SE)	p-value	Coefficients (SE)	p-value	N_case/N_total	Odds ratio (95% CI)	p-value	N_case/N_total	Odds ratio (95% CI)	p-value	N_case/N_total	Odds ratio (95% CI)	p-value
KDM-BAacc (Male)[b]	-0.0063 (0.0002)	< 0.0001	-0.0122 (0.0010)	< 0.0001	2152/10735	1.72 (1.56–1.89)	< 0.0001	2152/10735	1.3 (1.18–1.44)	< 0.0001	892/10735	1.65 (1.43–1.9)	< 0.0001
**KDM-BAacc (Quartile)**													
Q1	Ref		Ref		383/2684	Ref		444/2684	Ref		142/2684	Ref	
Q2	-0.0268 (0.0025)	< 0.0001	-0.0565 (0.0100)	< 0.0001	484/2684	1.37 (1.18–1.59)	< 0.0001	537/2684	1.3 (1.13–1.49)	0.0003	212/2684	1.59 (1.28–1.99)	< 0.0001
Q3	-0.0423 (0.0025)	< 0.0001	-0.0751 (0.0101)	< 0.0001	572/2684	1.76 (1.52–2.04)	< 0.0001	533/2684	1.33 (1.16–1.54)	0.0001	227/2684	1.78 (1.43–2.23)	< 0.0001
Q4	-0.0596 (0.0025)	< 0.0001	-0.1309 (0.0101)	< 0.0001	713/2683	2.33 (2.02–2.68)	< 0.0001	638/2683	1.66 (1.45–1.91)	< 0.0001	311/2683	2.51 (2.04–3.11)	< 0.0001
KDM-BAacc (Female)	-0.0045 (0.0001)	< 0.0001	-0.0082 (0.0004)	< 0.0001	3746/18702	1.99 (1.85–2.15)	< 0.0001	3746/18702	1.52 (1.41–1.64)	< 0.0001	1789/18702	2.07 (1.87–2.3)	< 0.0001
**KDM-BAacc (Quartile)**													
Q1	Ref		Ref		523/4676	Ref		707/4676	Ref		245/4676	Ref	
Q2	-0.0161 (0.0014)	< 0.0001	-0.0182 (0.0049)	0.0002	802/4676	1.59 (1.41–1.8)	< 0.0001	815/4676	1.14 (1.02–1.28)	0.0213	338/4676	1.32 (1.11–1.57)	0.0015
Q3	-0.0277 (0.0014)	< 0.0001	-0.0422 (0.0049)	< 0.0001	977/4675	1.97 (1.75–2.22)	< 0.0001	969/4675	1.39 (1.25–1.55)	< 0.0001	459/4675	1.81 (1.54–2.13)	< 0.0001
Q4	-0.0467 (0.0015)	< 0.0001	-0.0831 (0.0050)	< 0.0001	1444/4675	3.23 (2.88–3.62)	< 0.0001	1255/4675	1.86 (1.68–2.07)	< 0.0001	747/4675	3.03 (2.6–3.54)	< 0.0001

[a]Adjusted for age, socioeconomic status (residence, education level, marital status), lifestyle behaviors (smoking, drinking, physical activity)

[b]For ASMI and relative grip strength, KDM-BAacc was modeled as a continuous variable (per 1-year increase) in linear regression models. For LMM, LMS, and sarcopenia, KDM-BAacc was dichotomized (accelerated vs. non-accelerated aging) in logistic regression models, with all outcomes treated as binary variables.

TABLE S12. Associations between KDM-BAacc and muscle-related indicators (KDM based on Chinese biomarker set and NHANES-trained parameters, applied to the Chinese dataset[a],fully adjusted model[b])

Biological age	ASMI		Relative Grip Strength		LMM			LMS			Sarcopenia		
	Coefficients (SE)	p-value	Coefficients (SE)	p-value	N_case/N_total	Odds ratio (95% CI)	p-value	N_case/N_total	Odds ratio (95% CI)	p-value	N_case/N_total	Odds ratio (95% CI)	p-value
KDM-BAacc (Male)[c]	0.0004 (0.0003)	0.2256	-0.0086 (0.0011)	< 0.0001	2152/10735	1.01 (0.9–1.15)	0.8136	2152/10735	1.27 (1.13–1.43)	0.0001	892/10735	1.17 (0.99–1.39)	0.0623
KDM-BAacc (Quartile)													
Q1	Ref		Ref		500/2684	Ref		429/2684	Ref		168/2684	Ref	
Q2	0.0006 (0.0026)	0.8318	-0.0169 (0.0101)	0.0931	508/2684	0.95 (0.82–1.09)	0.4729	478/2684	1.05 (0.91–1.22)	0.5162	213/2684	1.15 (0.92–1.43)	0.2118
Q3	0.0033 (0.0027)	0.2236	-0.0333 (0.0105)	0.0014	531/2684	0.93 (0.81–1.08)	0.353	543/2684	1.13 (0.97–1.31)	0.1093	216/2684	1.05 (0.84–1.31)	0.6791
Q4	0.0025 (0.0029)	0.3958	-0.0680 (0.0113)	< 0.0001	613/2683	0.97 (0.83–1.13)	0.7087	702/2683	1.3 (1.11–1.52)	0.0009	295/2683	1.21 (0.96–1.52)	0.1056
KDM-BAacc (Female)	-0.0024 (0.0001)	< 0.0001	-0.0027 (0.0003)	< 0.0001	3746/18702	1.67 (1.54–1.81)	< 0.0001	3746/18702	1.23 (1.13–1.33)	< 0.0001	1789/18702	1.53 (1.38–1.71)	< 0.0001
KDM-BAacc (Quartile)													
Q1	Ref		Ref		622/4676	Ref		798/4676	Ref		292/4676	Ref	
Q2	-0.0103 (0.0015)	< 0.0001	-0.0032 (0.0050)	0.5254	751/4676	1.19 (1.06–1.34)	0.0042	825/4676	0.99 (0.89–1.11)	0.891	344/4676	1.09 (0.93–1.29)	0.2927
Q3	-0.0222 (0.0015)	< 0.0001	-0.0194 (0.0051)	0.0001	1001/4675	1.57 (1.4–1.77)	< 0.0001	973/4675	1.14 (1.02–1.27)	0.0172	469/4675	1.39 (1.18–1.63)	0.0001
Q4	-0.0339 (0.0016)	< 0.0001	-0.0366 (0.0053)	< 0.0001	1372/4675	2.15 (1.91–2.41)	< 0.0001	1150/4675	1.25 (1.12–1.4)	0.0001	684/4675	1.85 (1.58–2.17)	< 0.0001

[a]Due to missing biomarker variables in the NHANES dataset, three out of the 12 male-specific biomarkers—alanine aminotransferase, lymphocyte absolute count, and platelet count—were not available. Similarly, among the 13 female-specific biomarkers, alanine aminotransferase, aspartate aminotransferase, and platelet count were missing and thus excluded from the sensitivity analysis using NHANES-trained parameters

[b]Fully adjusted for age, socioeconomic status (residence, education level, marital status), lifestyle behaviors (smoking, drinking, physical activity), and clinical comorbidities (hypertension and diabetes)

[c]For ASMI and relative grip strength, KDM-BAacc was modeled as a continuous variable (per 1-year increase) in linear regression models. For LMM, LMS, and sarcopenia, KDM-BAacc was dichotomized (accelerated vs. non-accelerated aging) in logistic regression models, with all outcomes treated as binary variables.

TABLE S13. Associations between biological age accelerations and muscle-related indicators (KDM based on NHANES biomarker set and NHANES-trained parameters, applied to the Chinese dataset[a],fully adjusted model[b])

Biological age	ASMI		Relative Grip Strength		LMM			LMS			Sarcopenia		
	Coefficients (SE)	p-value	Coefficients (SE)	p-value	N_case/N_total	Odds ratio (95% CI)	p-value	N_case/N_total	Odds ratio (95% CI)	p-value	N_case/N_total	Odds ratio (95% CI)	p-value
KDM-BAacc (Male)[c]	-0.0024 (0.0004)	< 0.0001	-0.0089 (0.0014)	< 0.0001	2152/10735	1.31 (1.16–1.47)	< 0.0001	2152/10735	1.22 (1.08–1.37)	0.0013	892/10735	1.37 (1.15–1.61)	0.0003
KDM-BAacc (Quartile)													
Q1	Ref		Ref		435/2684	Ref		478/2684	Ref		170/2684	Ref	
Q2	-0.0086 (0.0026)	0.0011	0.0034 (0.0101)	0.7357	499/2684	1.12 (0.97–1.3)	0.1258	470/2684	0.87 (0.75–1.01)	0.0645	193/2684	0.99 (0.79–1.23)	0.9049
Q3	-0.0119 (0.0028)	< 0.0001	-0.0173 (0.0107)	0.1062	536/2684	1.17 (1.01–1.36)	0.043	534/2684	0.93 (0.8–1.08)	0.3551	224/2684	1.04 (0.83–1.3)	0.7402
Q4	-0.0193 (0.0030)	< 0.0001	-0.0566 (0.0116)	< 0.0001	682/2683	1.46 (1.25–1.71)	< 0.0001	670/2683	1.06 (0.91–1.24)	0.4469	305/2683	1.23 (0.98–1.55)	0.0726
KDM-BAacc (Female)	-0.0047 (0.0003)	< 0.0001	-0.0039 (0.0009)	< 0.0001	3746/18702	1.48 (1.36–1.61)	< 0.0001	3746/18702	1.15 (1.06–1.25)	0.0013	1789/18702	1.40 (1.26–1.57)	< 0.0001
KDM-BAacc (Quartile)													
Q1	Ref		Ref		637/4676	Ref		800/4676	Ref		310/4676	Ref	
Q2	-0.0087 (0.0015)	< 0.0001	0.0001 (0.0050)	0.9795	791/4676	1.22 (1.08–1.37)	0.001	862/4676	1.03 (0.93–1.15)	0.5642	357/4676	1.06 (0.9–1.24)	0.513
Q3	-0.0163 (0.0015)	< 0.0001	-0.0080 (0.0051)	0.1161	985/4675	1.46 (1.3–1.63)	< 0.0001	942/4675	1.07 (0.96–1.2)	0.2127	464/4675	1.25 (1.06–1.46)	0.0065
Q4	-0.0258 (0.0016)	< 0.0001	-0.0218 (0.0054)	1e-04	1333/4675	1.86 (1.65–2.09)	< 0.0001	1142/4675	1.19 (1.07–1.34)	0.0023	658/4675	1.54 (1.31–1.8)	< 0.0001

[a]The NHANES-based KDM model included nine biomarkers: total cholesterol, systolic blood pressure, creatinine, blood urea nitrogen, albumin, alkaline phosphatase, C-reactive protein, glycohemoglobin, and forced expiratory volume in 1 second (FEV_1). Due to missing data on C-reactive protein, glycohemoglobin, and FEV_1 in our dataset, only the remaining six biomarkers were used in the sensitivity analysis.

[b]Fully adjusted for age, socioeconomic status (residence, education level, marital status), lifestyle behaviors (smoking, drinking, physical activity), and clinical comorbidities (hypertension and diabetes)

[c]For ASMI and relative grip strength, biological age acceleration (KDM-BAacc) was modeled as a continuous variable (per 1-year increase) in linear regression models. For LMM, LMS, and sarcopenia, KDM-BAacc was dichotomized (accelerated vs. non-accelerated aging) in logistic regression models, with all outcomes treated as binary variable

TABLE S14. Associations between KDM-BAacc and muscle-related indicators (additional adjustment for socioeconomic status (SES), categorized into three groups)[a]

Biological age	ASMI		Relative Grip Strength		LMM			LMS			Sarcopenia		
	Coefficients (SE)	p-value	Coefficients (SE)	p-value	N_case/N_total	Odds ratio (95% CI)	p-value	N_case/N_total	Odds ratio (95% CI)	p-value	N_case/N_total	Odds ratio (95% CI)	p-value
KDM-BAacc (Male)[b]	-0.0064 (0.0002)	< 0.0001	-0.0133 (0.0010)	< 0.0001	2152/10735	1.73 (1.57–1.91)	< 0.0001	2152/10735	1.34 (1.22–1.48)	< 0.0001	892/10735	1.71 (1.48–1.98)	< 0.0001
KDM-BAacc (Quartile)													
Q1	Ref		Ref		383/2684	Ref		444/2684	Ref		142/2684	Ref	
Q2	-0.0277 (0.0025)	< 0.0001	-0.0635 (0.0099)	< 0.0001	484/2684	1.41 (1.21–1.64)	< 0.0001	537/2684	1.35 (1.17–1.56)	1e-04	212/2684	1.65 (1.32–2.08)	< 0.0001
Q3	-0.0432 (0.0025)	< 0.0001	-0.0829 (0.0100)	< 0.0001	572/2684	1.81 (1.56–2.1)	< 0.0001	533/2684	1.39 (1.21–1.61)	< 0.0001	227/2684	1.89 (1.52–2.38)	< 0.0001
Q4	-0.0605 (0.0025)	< 0.0001	-0.1402 (0.0100)	< 0.0001	713/2683	2.38 (2.06–2.75)	< 0.0001	638/2683	1.76 (1.53–2.03)	< 0.0001	311/2683	2.65 (2.14–3.29)	< 0.0001
KDM-BAacc (Female)	-0.0041 (0.0001)	< 0.0001	-0.0065 (0.0005)	< 0.0001	3746/18702	1.77 (1.64–1.92)	< 0.0001	3746/18702	1.38 (1.27–1.49)	< 0.0001	1789/18702	1.8 (1.61–2.01)	< 0.0001
KDM-BAacc (Quartile)													
Q1	Ref		Ref		523/4676	Ref		707/4676	Ref		245/4676	Ref	
Q2	-0.0150 (0.0015)	< 0.0001	-0.0143 (0.0050)	0.0043	802/4676	1.55 (1.37–1.76)	< 0.0001	815/4676	1.1 (0.98–1.24)	0.0897	338/4676	1.27 (1.07–1.52)	0.0076
Q3	-0.0252 (0.0015)	< 0.0001	-0.0339 (0.0051)	< 0.0001	977/4675	1.85 (1.64–2.09)	< 0.0001	969/4675	1.31 (1.17–1.47)	< 0.0001	459/4675	1.67 (1.41–1.98)	< 0.0001
Q4	-0.0419 (0.0016)	< 0.0001	-0.0644 (0.0054)	< 0.0001	1444/4675	2.84 (2.51–3.21)	< 0.0001	1255/4675	1.62 (1.44–1.82)	< 0.0001	747/4675	2.53 (2.15–2.99)	< 0.0001

[a]fully adjusted for age, residence, education level, marital status, lifestyle behaviors (smoking, drinking, physical activity), clinical comorbidities (hypertension and diabetes), and and socioeconomic status (SES), categorized into three groups (SES3).

[b]For ASMI and relative grip strength, KDM-BAacc was modeled as a continuous variable (per 1-year increase) in linear regression models. For LMM, LMS, and sarcopenia, KDM-BAacc was dichotomized (accelerated vs. non-accelerated aging) in logistic regression models, with all outcomes treated as binary variables.

TABLE S15. Associations between KDM-BAacc and muscle-related indicators (additional adjustment for socioeconomic status (SES), categorized into four groups)[a]

Biological age	ASMI		Relative Grip Strength		LMM			LMS			Sarcopenia		
	Coefficients (SE)	p-value	Coefficients (SE)	p-value	N_case/N_total	Odds ratio (95% CI)	p-value	N_case/N_total	Odds ratio (95% CI)	p-value	N_case/N_total	Odds ratio (95% CI)	p-value
KDM-BAacc (Male)[b]	-0.0064 (0.0002)	< 0.0001	-0.0133 (0.0010)	< 0.0001	2152/10735	1.73 (1.57–1.91)	< 0.0001	2152/10735	1.34 (1.22–1.48)	< 0.0001	892/10735	1.71 (1.48–1.98)	< 0.0001
**KDM-BAacc (Quartile)**													
Q1	Ref		Ref		383/2684	Ref		444/2684	Ref		142/2684	Ref	
Q2	-0.0277 (0.0025)	< 0.0001	-0.0637 (0.0099)	< 0.0001	484/2684	1.41 (1.21–1.64)	< 0.0001	537/2684	1.35 (1.17–1.56)	< 0.0001	212/2684	1.66 (1.32–2.08)	< 0.0001
Q3	-0.0432 (0.0025)	< 0.0001	-0.0827 (0.0100)	< 0.0001	572/2684	1.81 (1.56–2.1)	< 0.0001	533/2684	1.39 (1.21–1.61)	< 0.0001	227/2684	1.89 (1.52–2.38)	< 0.0001
Q4	-0.0604 (0.0025)	< 0.0001	-0.1401 (0.0100)	< 0.0001	713/2683	2.38 (2.06–2.75)	< 0.0001	638/2683	1.76 (1.53–2.03)	< 0.0001	311/2683	2.65 (2.14–3.29)	< 0.0001
KDM-BAacc (Female)	-0.0041 (0.0001)	< 0.0001	-0.0065 (0.0005)	< 0.0001	3746/18702	1.77 (1.63–1.92)	< 0.0001	3746/18702	1.38 (1.27–1.49)	< 0.0001	1789/18702	1.79 (1.6–2.01)	< 0.0001
**KDM-BAacc (Quartile)**													
Q1	Ref		Ref		523/4676	Ref		707/4676	Ref		245/4676	Ref	
Q2	-0.0150 (0.0015)	< 0.0001	-0.0141 (0.0050)	0.0047	802/4676	1.55 (1.37–1.75)	< 0.0001	815/4676	1.1 (0.98–1.23)	0.0968	338/4676	1.27 (1.06–1.51)	0.0085
Q3	-0.0252 (0.0015)	< 0.0001	-0.0336 (0.0051)	< 0.0001	977/4675	1.85 (1.64–2.09)	< 0.0001	969/4675	1.31 (1.17–1.47)	< 0.0001	459/4675	1.66 (1.41–1.97)	< 0.0001
Q4	-0.0419 (0.0016)	< 0.0001	-0.0642 (0.0054)	< 0.0001	1444/4675	2.83 (2.51–3.2)	< 0.0001	1255/4675	1.62 (1.44–1.81)	< 0.0001	747/4675	2.52 (2.14–2.98)	< 0.0001

[a]fully adjusted for age, residence, education level, marital status, lifestyle behaviors (smoking, drinking, physical activity), clinical comorbidities (hypertension and diabetes), and and socioeconomic status (SES), categorized into four groups (SES4).

[b]For ASMI and relative grip strength, KDM-BAacc was modeled as a continuous variable (per 1-year increase) in linear regression models. For LMM, LMS, and sarcopenia, KDM-BAacc was dichotomized (accelerated vs. non-accelerated aging) in logistic regression models, with all outcomes treated as binary variables.

TABLE S16. Sex- and socioeconomic status–stratified associations between KDM-BA acceleration and muscle-related outcomes (categorized into three groups)[a]

Strata		LMM			LMS			Sarcopenia		
Sex	SES3	N_case/N_total	Odds ratio (95% CI)	p-value	N_case/N_total	Odds ratio (95% CI)	p-value	N_case/N_total	Odds ratio (95% CI)	p-value
Male	Low	870/3791	1.86 (1.59–2.17)	< 0.0001	880/3791	1.41 (1.21–1.65)	< 0.0001	376/3791	1.89 (1.51–2.36)	< 0.0001
Male	Medium	867/4366	1.59 (1.36–1.86)	< 0.0001	804/4366	1.29 (1.10–1.52)	0.0016	329/4366	1.55 (1.23–1.97)	0.0002
Male	High	356/2326	1.82 (1.43–2.31)	< 0.0001	416/2326	1.30 (1.04–1.62)	0.0226	158/2326	1.66 (1.18–2.36)	0.0040
Female	Low	1906/6902	1.55 (1.38–1.75)	< 0.0001	1813/6902	1.26 (1.12–1.41)	0.0002	959/6902	1.59 (1.36–1.85)	< 0.0001
Female	Medium	1268/7750	1.87 (1.64–2.13)	< 0.0001	1289/7750	1.44 (1.27–1.64)	< 0.0001	565/7750	1.82 (1.51–2.20)	< 0.0001
Female	High	464/3575	2.34 (1.89–2.90)	< 0.0001	541/3575	1.66 (1.36–2.02)	< 0.0001	209/3575	2.72 (1.99–3.73)	< 0.0001

[a]fully adjusted for age, residence, education level, marital status, lifestyle behaviors (smoking, drinking, physical activity), and clinical comorbidities (hypertension and diabetes)
[b]For LMM, LMS, and sarcopenia, KDM-BAacc was dichotomized (accelerated vs. non-accelerated aging) in logistic regression models, with all outcomes treated as binary variables.

TABLE S17. Sex- and socioeconomic status–stratified associations between KDM-BA acceleration and muscle-related outcomes (categorized into four groups)[a]

Strata		LMM			LMS			Sarcopenia		
Sex	SES4	N_case/N_total	Odds ratio (95% CI)	p-value	N_case/N_total	Odds ratio (95% CI)	p-value	N_case/N_total	Odds ratio (95% CI)	p-value
Female	Low	1824/6596	1.57 (1.39–1.77)	< 0.0001	1746/6596	1.25 (1.11–1.41)	0.0003	924/6596	1.60 (1.36–1.88)	< 0.0001
Female	Medium-Low	258/1193	1.89 (1.37–2.60)	< 0.0001	218/1193	1.92 (1.38–2.68)	0.0001	110/1193	1.67 (1.06–2.66)	0.0282
Female	Medium-High	1080/6719	1.82 (1.58–2.10)	< 0.0001	1118/6719	1.37 (1.19–1.57)	< 0.0001	485/6719	1.79 (1.47–2.20)	< 0.0001
Female	High	476/3719	2.32 (1.88–2.86)	< 0.0001	561/3719	1.63 (1.35–1.98)	< 0.0001	214/3719	2.60 (1.92–3.54)	< 0.0001
Male	Low	805/3541	1.90 (1.62–2.24)	< 0.0001	823/3541	1.38 (1.18–1.63)	< 0.0001	351/3541	1.85 (1.47–2.34)	< 0.0001
Male	Medium-Low	234/1023	1.90 (1.40–2.58)	< 0.0001	190/1023	1.60 (1.15–2.23)	0.0055	84/1023	2.33 (1.45–3.83)	0.0006
Male	Medium-High	676/3460	1.48 (1.24–1.76)	< 0.0001	643/3460	1.23 (1.03–1.47)	0.0224	262/3460	1.41 (1.08–1.83)	0.0108
Male	High	378/2459	1.84 (1.46–2.32)	< 0.0001	444/2459	1.33 (1.07–1.65)	0.0099	166/2459	1.70 (1.22–2.40)	0.0019

[a]fully adjusted for age, residence, education level, marital status, lifestyle behaviors (smoking, drinking, physical activity), and clinical comorbidities (hypertension and diabetes)
[b]For LMM, LMS, and sarcopenia, KDM-BAacc was dichotomized (accelerated vs. non-accelerated aging) in logistic regression models, with all outcomes treated as binary variables.

TABLE S18. Associations between KDM-BAacc and muscle-related indicators (additional adjustment for dietary status)[a]

Biological age	ASMI		Relative Grip Strength		LMM			LMS			Sarcopenia		
	Coefficients (SE)	p-value	Coefficients (SE)	p-value	N_case/N_total	Odds ratio (95% CI)	p-value	N_case/N_total	Odds ratio (95% CI)	p-value	N_case/N_total	Odds ratio (95% CI)	p-value
KDM-BAacc (Male)[b]	-0.0063 (0.0002)	< 0.0001	-0.0130 (0.0010)	< 0.0001	2152/10735	1.73 (1.56–1.91)	< 0.0001	2152/10735	1.33 (1.21–1.47)	< 0.0001	892/10735	1.7 (1.47–1.96)	< 0.0001
**KDM-BAacc (Quartile)**													
Q1	Ref		Ref		383/2684	Ref		444/2684	Ref		142/2684	Ref	
Q2	-0.0274 (0.0025)	< 0.0001	-0.0616 (0.0099)	< 0.0001	484/2684	1.4 (1.2–1.63)	< 0.0001	537/2684	1.34 (1.16–1.54)	0.0001	212/2684	1.63 (1.3–2.05)	< 0.0001
Q3	-0.0429 (0.0025)	< 0.0001	-0.0803 (0.0100)	< 0.0001	572/2684	1.8 (1.56–2.09)	< 0.0001	533/2684	1.38 (1.2–1.6)	< 0.0001	227/2684	1.88 (1.5–2.35)	< 0.0001
Q4	-0.0599 (0.0025)	< 0.0001	-0.1367 (0.0100)	< 0.0001	713/2683	2.35 (2.04–2.72)	< 0.0001	638/2683	1.74 (1.51–2)	< 0.0001	311/2683	2.6 (2.1–3.23)	< 0.0001
KDM-BAacc (Female)	-0.0041 (0.0001)	< 0.0001	-0.0064 (0.0005)	< 0.0001	3746/18702	1.77 (1.64–1.92)	< 0.0001	3746/18702	1.38 (1.27–1.49)	< 0.0001	1789/18702	1.8 (1.61–2.01)	< 0.0001
**KDM-BAacc (Quartile)**													
Q1	Ref		Ref		523/4676	Ref		707/4676	Ref		245/4676	Ref	
Q2	-0.0148 (0.0015)	< 0.0001	-0.0135 (0.0050)	0.0066	802/4676	1.54 (1.36–1.75)	< 0.0001	815/4676	1.1 (0.98–1.23)	0.1082	338/4676	1.26 (1.06–1.51)	0.0091
Q3	-0.0249 (0.0015)	< 0.0001	-0.0330 (0.0051)	< 0.0001	977/4675	1.84 (1.63–2.08)	< 0.0001	969/4675	1.3 (1.17–1.46)	< 0.0001	459/4675	1.66 (1.4–1.97)	< 0.0001
Q4	-0.0417 (0.0016)	< 0.0001	-0.0637 (0.0054)	< 0.0001	1444/4675	2.83 (2.51–3.2)	< 0.0001	1255/4675	1.61 (1.44–1.81)	< 0.0001	747/4675	2.53 (2.14–2.99)	< 0.0001

[a]fully adjusted for age, residence, education level, marital status, lifestyle behaviors (smoking, drinking, physical activity), clinical comorbidities (hypertension and diabetes), and dietary status.

[b]For ASMI and relative grip strength, KDM-BAacc was modeled as a continuous variable (per 1-year increase) in linear regression models. For LMM, LMS, and sarcopenia, KDM-BAacc was dichotomized (accelerated vs. non-accelerated aging) in logistic regression models, with all outcomes treated as binary variables.

TABLE S19. Associations between KDM-BAacc and muscle-related indicators (additional adjustment for pharmaceutical usage)[a]

Biological age	ASMI		Relative Grip Strength		LMM			LMS			Sarcopenia		
	Coefficients (SE)	p-value	Coefficients (SE)	p-value	N_case/N_total	Odds ratio (95% CI)	p-value	N_case/N_total	Odds ratio (95% CI)	p-value	N_case/N_total	Odds ratio (95% CI)	p-value
KDM-BAacc (Male)[b]	-0.0064 (0.0002)	< 0.0001	-0.0127 (0.0010)	< 0.0001	2152/10735	1.74 (1.57–1.92)	< 0.0001	2152/10735	1.32 (1.2–1.46)	< 0.0001	892/10735	1.67 (1.45–1.93)	< 0.0001
**KDM-BAacc (Quartile)**													
Q1	Ref		Ref		383/2684	Ref		444/2684	Ref		142/2684	Ref	
Q2	-0.0281 (0.0025)	< 0.0001	-0.0637 (0.0098)	< 0.0001	484/2684	1.4 (1.21–1.63)	< 0.0001	537/2684	1.35 (1.17–1.56)	< 0.0001	212/2684	1.65 (1.32–2.06)	< 0.0001
Q3	-0.0434 (0.0025)	< 0.0001	-0.0796 (0.0099)	< 0.0001	572/2684	1.8 (1.56–2.08)	< 0.0001	533/2684	1.37 (1.19–1.59)	< 0.0001	227/2684	1.83 (1.47–2.29)	< 0.0001
Q4	-0.0609 (0.0025)	< 0.0001	-0.1362 (0.0099)	< 0.0001	713/2683	2.4 (2.08–2.77)	< 0.0001	638/2683	1.73 (1.5–1.99)	< 0.0001	311/2683	2.61 (2.11–3.23)	< 0.0001
KDM-BAacc (Female)	-0.0042 (0.0001)	< 0.0001	-0.0065 (0.0005)	< 0.0001	3746/18702	1.77 (1.63–1.92)	< 0.0001	3746/18702	1.37 (1.27–1.49)	< 0.0001	1789/18702	1.78 (1.6–1.99)	< 0.0001
**KDM-BAacc (Quartile)**													
Q1	Ref		Ref		523/4676	Ref		707/4676	Ref		245/4676	Ref	
Q2	-0.0151 (0.0014)	< 0.0001	-0.0145 (0.0049)	0.0033	802/4676	1.54 (1.36–1.74)	< 0.0001	815/4676	1.11 (0.99–1.24)	0.0735	338/4676	1.27 (1.07–1.51)	0.0071
Q3	-0.0251 (0.0015)	< 0.0001	-0.0337 (0.0050)	< 0.0001	977/4675	1.83 (1.62–2.06)	< 0.0001	969/4675	1.31 (1.17–1.46)	< 0.0001	459/4675	1.64 (1.39–1.94)	< 0.0001
Q4	-0.0419 (0.0015)	< 0.0001	-0.0639 (0.0053)	< 0.0001	1444/4675	2.82 (2.5–3.18)	< 0.0001	1255/4675	1.61 (1.44–1.81)	< 0.0001	747/4675	2.51 (2.13–2.96)	< 0.0001

[a]fully adjusted for age, residence, education level, marital status, lifestyle behaviors (smoking, drinking, physical activity), clinical comorbidities (hypertension and diabetes), and pharmaceutical usage.

[b]For ASMI and relative grip strength, KDM-BAacc was modeled as a continuous variable (per 1-year increase) in linear regression models. For LMM, LMS, and sarcopenia, KDM-BAacc was dichotomized (accelerated vs. non-accelerated aging) in logistic regression models, with all outcomes treated as binary variables.

TABLE S20. Sex-stratified multivariable logistic regression analyses of factors associated with aging, LMM, LMS, and sarcopenia[a]

Determinants	aging				LMM			
	Male	p	Female	p	Male	p	Female	p
Alcohol: Current	0.97 (0.89–1.05)	0.4508	1.09 (0.97–1.21)	0.1364	0.99 (0.89–1.11)	0.9132	1.24 (1.08–1.41)	0.0019
Alcohol: Former	0.87 (0.75–1.01)	0.0631	0.94 (0.70–1.26)	0.6664	1.16 (0.97–1.39)	0.1017	1.46 (1.04–2.02)	0.0238
Drug use: No	0.79 (0.73–0.86)	< 0.0001	0.53 (0.49–0.56)	< 0.0001	0.85 (0.77–0.95)	0.0023	0.84 (0.78–0.92)	< 0.0001
Education: College or above	1.14 (0.97–1.34)	0.1221	0.65 (0.58–0.74)	< 0.0001	0.78 (0.64–0.96)	0.0223	0.57 (0.48–0.68)	< 0.0001
Education: High school or equivalent	1.11 (0.98–1.24)	0.0925	0.84 (0.76–0.92)	0.0002	0.89 (0.77–1.03)	0.1071	0.81 (0.71–0.91)	0.0005
Exercise: 1–2/wk	1.29 (1.12–1.48)	0.0005	1.00 (0.90–1.11)	0.9835	0.93 (0.78–1.11)	0.4287	0.95 (0.84–1.08)	0.4401
Exercise: 3–4/wk	1.09 (0.93–1.27)	0.2861	0.91 (0.81–1.02)	0.0953	0.81 (0.67–0.99)	0.0401	0.83 (0.71–0.96)	0.0104
Exercise: 5–7/wk	1.14 (1.03–1.26)	0.0099	0.97 (0.90–1.05)	0.4788	0.77 (0.68–0.87)	< 0.0001	0.67 (0.61–0.73)	< 0.0001
Exercise: ≤ 3/mo	1.42 (1.21–1.68)	< 0.0001	1.06 (0.94–1.19)	0.3660	1.01 (0.83–1.22)	0.9542	1.02 (0.88–1.18)	0.7500
Healthy diet score: 3–5	0.94 (0.82–1.07)	0.3519	0.97 (0.86–1.08)	0.5481	0.74 (0.64–0.86)	< 0.0001	0.76 (0.67–0.86)	< 0.0001
Healthy diet score: 6–8	0.86 (0.74–1.00)	0.0575	0.92 (0.81–1.04)	0.1746	0.58 (0.49–0.70)	< 0.0001	0.61 (0.53–0.70)	< 0.0001
Marital status: Divorced	1.05 (0.80–1.37)	0.7329	0.85 (0.70–1.04)	0.1099	1.25 (0.90–1.70)	0.1713	0.91 (0.69–1.18)	0.4835
Marital status: Never married	0.73 (0.61–0.86)	0.0003	1.48 (1.28–1.71)	< 0.0001	1.49 (1.21–1.83)	0.0001	1.24 (1.02–1.51)	0.0320
Marital status: Widowed	0.96 (0.75–1.24)	0.7565	0.68 (0.61–0.77)	< 0.0001	1.11 (0.82–1.49)	0.4802	1.19 (1.04–1.36)	0.0129
Residence: Rural	0.93 (0.84–1.03)	0.1508	1.12 (1.04–1.21)	0.0032	1.00 (0.88–1.12)	0.9470	1.26 (1.15–1.38)	< 0.0001
SES: High	1.07 (0.88–1.29)	0.5065	0.99 (0.85–1.14)	0.8495	0.80 (0.62–1.02)	0.0678	0.86 (0.70–1.06)	0.1607
SES: Middle	1.15 (1.03–1.30)	0.0175	0.94 (0.86–1.03)	0.1880	0.97 (0.84–1.12)	0.6877	0.80 (0.71–0.89)	< 0.0001
Smoking: Current	1.44 (1.32–1.58)	< 0.0001	0.72 (0.60–0.88)	0.0008	0.87 (0.77–0.97)	0.0121	0.70 (0.55–0.88)	0.0031
Smoking: Former	1.14 (1.02–1.28)	0.0271	0.78 (0.54–1.12)	0.1798	0.90 (0.78–1.04)	0.1548	1.07 (0.70–1.60)	0.7480

Determinants	LMS				Sarcopenia			
	Male	p	Female	p	Male	p	Female	p
Alcohol: Current	0.93 (0.84–1.04)	0.2095	1.11 (0.96–1.26)	0.1466	1.00 (0.86–1.18)	0.9566	1.24 (1.03–1.48)	0.0187
Alcohol: Former	1.26 (1.05–1.50)	0.0112	1.71 (1.24–2.34)	0.0008	1.26 (0.97–1.62)	0.0787	2.21 (1.50–3.19)	< 0.0001
Drug use: No	0.58 (0.53–0.65)	< 0.0001	0.76 (0.70–0.83)	< 0.0001	0.69 (0.59–0.79)	< 0.0001	0.80 (0.71–0.89)	< 0.0001
Education: College or above	1.03 (0.84–1.27)	0.7557	0.77 (0.65–0.91)	0.0025	1.04 (0.77–1.40)	0.7888	0.65 (0.50–0.83)	0.0007
Education: High school or equivalent	0.76 (0.65–0.89)	0.0005	0.90 (0.80–1.02)	0.0913	0.85 (0.68–1.06)	0.1625	0.91 (0.76–1.07)	0.2531
Exercise: 1–2/wk	0.90 (0.75–1.07)	0.2282	0.69 (0.61–0.79)	< 0.0001	0.93 (0.73–1.19)	0.5775	0.73 (0.61–0.88)	0.0008
Exercise: 3–4/wk	0.70 (0.57–0.86)	0.0005	0.74 (0.65–0.86)	< 0.0001	0.72 (0.54–0.96)	0.0264	0.79 (0.65–0.96)	0.0170
Exercise: 5–7/wk	0.66 (0.58–0.75)	< 0.0001	0.61 (0.56–0.67)	< 0.0001	0.63 (0.52–0.75)	< 0.0001	0.59 (0.52–0.67)	< 0.0001
Exercise: ≤ 3/mo	1.03 (0.85–1.24)	0.7725	0.73 (0.63–0.84)	< 0.0001	0.97 (0.74–1.27)	0.8431	0.87 (0.71–1.05)	0.1529
Healthy diet score: 3–5	0.77 (0.66–0.90)	0.0008	0.79 (0.70–0.90)	0.0003	0.72 (0.59–0.89)	0.0019	0.67 (0.58–0.79)	< 0.0001
Healthy diet score: 6–8	0.63 (0.52–0.75)	< 0.0001	0.69 (0.60–0.80)	< 0.0001	0.51 (0.39–0.67)	< 0.0001	0.56 (0.47–0.68)	< 0.0001
Marital status: Divorced	1.43 (1.04–1.94)	0.0237	1.09 (0.85–1.38)	0.4893	1.59 (1.03–2.36)	0.0287	1.07 (0.75–1.49)	0.6964
Marital status: Never married	1.65 (1.34–2.01)	< 0.0001	1.29 (1.06–1.55)	0.0092	2.02 (1.54–2.63)	< 0.0001	1.55 (1.19–2.01)	0.0010
Marital status: Widowed	1.22 (0.90–1.62)	0.1912	0.89 (0.77–1.03)	0.1337	1.03 (0.65–1.55)	0.9027	0.96 (0.79–1.16)	0.6987
Residence: Rural	1.13 (1.00–1.27)	0.0549	1.10 (1.00–1.21)	0.0500	1.10 (0.92–1.31)	0.2905	1.19 (1.05–1.36)	0.0061
SES: High	0.81 (0.64–1.04)	0.0941	0.78 (0.64–0.95)	0.0128	0.72 (0.50–1.02)	0.0610	0.73 (0.55–0.97)	0.0305
SES: Middle	0.94 (0.81–1.09)	0.3952	0.74 (0.66–0.83)	< 0.0001	0.90 (0.73–1.11)	0.3232	0.71 (0.60–0.82)	< 0.0001
Smoking: Current	0.80 (0.71–0.90)	0.0001	0.85 (0.67–1.07)	0.1720	0.80 (0.68–0.94)	0.0082	0.69 (0.49–0.94)	0.0255
Smoking: Former	0.97 (0.84–1.12)	0.7043	1.06 (0.69–1.59)	0.7756	0.90 (0.73–1.12)	0.3476	0.84 (0.46–1.43)	0.5463

[a]For categorical covariates, odds ratios (ORs) were estimated relative to predefined reference categories, including urban residence, less than high school education, married status, never smoking, never drinking, no exercise, low socioeconomic status, lowest diet score (0–2), and drug use.

Articles published in the Biology of Sport are licensed under an open access Creative Commons CC BY 4.0 license.
